# Integration of In Vitro and In Silico Results From Chemical and Biological Assays of *Rheum turkestanicum* and *Calendula officinalis* Flower Extracts

**DOI:** 10.1002/fsn3.4663

**Published:** 2024-12-19

**Authors:** Serdar Korpayev, Gokhan Zengin, Gunes Ak, Jasmina Glamočlija, Marina Soković, Neda Aničić, Uroš Gašić, Dejan Stojković, Mirap Agamyradov, Mehmet Veysi Cetiz, Guljan Agamyradova

**Affiliations:** ^1^ Biotechnology Institute Ankara University Ankara Turkey; ^2^ Department of Biology, Science Faculty Selcuk University Konya Turkey; ^3^ Department of Plant Physiology, Institute for Biological Research “Siniša Stanković” – National Institute of Republic of Serbia University of Belgrade Belgrade Serbia; ^4^ Saint Petersburg State Pediatric Medical University St Petersburg Russia; ^5^ Cetiz Lab. Sanlıurfa Turkey; ^6^ Department of Chemistry Recep Tayyip Erdogan University Rize Turkey

**Keywords:** antibacterial, antifungal, antioxidant, *Calendula officinalis*, LC–MS, *Rheum turkestanicum*

## Abstract

In this study, we conducted a thorough analysis of *Rheum turkestanicum* (RT) and *Calendula officinalis flowers* (COF) extracts with varying polarities using LC–MS chemical profiling and biological tests (antioxidant, antimicrobial, enzyme inhibition, and cytotoxic effects). The highest level of total phenolic content in the ethanol extract of RT with 75.82 mg GAE/g, followed by the infusions of RT (65.00 mg GAE/g) and COF (40.99 mg GAE/g). A total of 20 bioactive compounds were identified and quantified. The ethanol extract of COF was rich in terms of 5‐O‐caffeoylquinic acid (2780.56 μg/g), isorhamnetin‐O‐rutinoside (1653.59 μg/g), and rutin (1356.97 μg/g). However, RF extracts were rich in catechin gallate (21.66–80.01 μg/g) and 5‐O‐caffeoylquinic acid. Except for metal chelating ability, the ethanol extract of RT exhibited the strongest ability (DPPH: 171.5 mg TE/g; ABTS: 387.35 mg TE/g; CUPRAC: 449.80 mg TE/g; FRAP: 195.60 mg TE/g; and PBD: 1.52 mmol TE/g). In the enzyme inhibition tests, the tested ethanol extracts for both species were more active than the infusion. The highest values for tyrosinase were recorded as 72.47 mg KAE/g (in RT extracts) and 71.74 mg KAE/g (in COF extracts). Furthermore, all extracts underwent assessment for their antibacterial and antifungal properties, targeting both Gram‐positive and Gram‐negative bacteria, as well as clinical yeast and fungal microorganisms. In silico studies yielded valuable insights into the potential therapeutic applications of the bioactive compounds identified in COF and RT extracts. Stable interactions were observed between key compounds, such as isorhamnetin 3‐O‐glucoside and 3‐O‐caffeoylquinic acid, with crucial target proteins (AChE, BChE, and MurE). These compounds formed stable hydrogen bonds with minimal root mean square deviation (RMSD) fluctuations, particularly in the isorhamnetin 3‐O‐glucoside‐ *Staphylococcus aureus* MurE and 3‐O‐caffeoylquinic acid‐MurE of *S. aureus* complexes. These findings further underscore the potential of these compounds as promising candidates for therapeutic development.

## Graphical abstract

The ethanol extract of *Rheum turkestanicum* contained the highest level of total phenolic. 5‐O‐caffeoylquinic acid and catechin gallate were detected as the main components. Good interactions were found between isorhamnetin 3‐O‐glucoside‐ *Staphylococcus aureus* MurE and 3‐O‐caffeoylquinic acid‐MurE of *S. aureus* complexes.

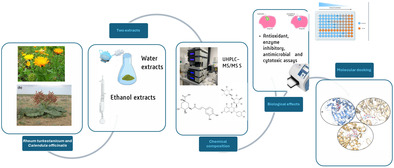

## 1 Introduction

A plant species known as *Rheum turkestanicum* (RT) is a member of the Polygonaceae family, also referred to as the rhubarb family (Amiri and Joharchi 2013; Taheri and Assadi 2013). This perennial herbaceous plant is indigenous to Central Asia (CA), including Turkestan, Tajikistan, Afghanistan, Iran, and parts of Turkmenistan (Rajaei et al. 2017). The plant is particularly notable for its high concentration of anthraquinone derivatives including emodin, aloe‐emodin, rhein, chrysophanol, danthron, and physcion. Other components that have been identified from rhubarb include vitamins, organic acids, dianthrones, stilbenes, anthocynins, falvonoids, anthraglycosides, and polyphenols (Zhang and Liu 2004). Therefore, the plant's high concentration of anthraquinones, which have potent laxative effects, has been utilized traditionally for therapeutic purposes (Ghorbani, Amiri, and Hosseini 2019). *R. turkestanicum* has a long history of usage in traditional medicine. This plant's rhizomes and roots have been used in traditional medicine to treat a variety of conditions, such as fever, liver issues, and digestive problems (Mohtashami et al. 2023). Additionally, studies have shown that *R. turkestanicum* is effective in the treatment of cancer, hypertension, and diabetes (Boroushaki et al. 2019; Moradzadeh et al. 2019). *R. turkestanicum* has a number of other uses in addition to its therapeutic capabilities. Yellow dye has been made from the plant's roots and used to color textiles and other items (Batsatsashvili, Kikvidze, and Bussmann 2020).

*Calendula officinalis* belongs to the Asteraceae family and is a species of plant that is also popularly known as marigold (Karimi Ansari and Koksal 2023). It is a short‐lived perennial herb that normally grows to a height of 30–60 cm. Its distinctive features include bright orange or yellow flowers that have a diameter of 4–7 cm and green, ovate leaves (Arora, Rani, and Sharma 2013; Bokelmann 2021). *C. officinalis* has been used traditionally in medicine to treat a wide range of diseases, such as wounds, skin irritations, digestive problems, and inflammation. Due to its antibacterial and anti‐inflammatory qualities, it is a well‐liked option for topical use (Shahane et al. 2023). In addition, *C. officinalis* is used in foods as a natural coloring and flavoring agent due to its soothing properties for the skin (Mur et al. 2021). The plant's chemical makeup includes flavonoids, triterpenoids, and essential oils, which are mostly in charge of its medicinal properties (Roy et al. 2022).

To the best of our knowledge, no thorough literature review has been conducted on the pharmacological properties and traditional medicinal uses of the *R. turkestanicum* (RT) and *C. officinalis* (COF) plants from Turkmenistan (Central Asia). Despite the existing knowledge about the morphological characteristics of these plants, comprehensive reviews focusing on the pharmacological properties and traditional medicinal uses of RT and COF in the context of Turkmenistan and Central Asia are limited. The aim of this study is to explore the phytochemistry and pharmacological properties of these plants that go beyond their physical descriptions.

## 2 Materials and Methods

### 2.1 Plant Materials

**FIGURE 1:**
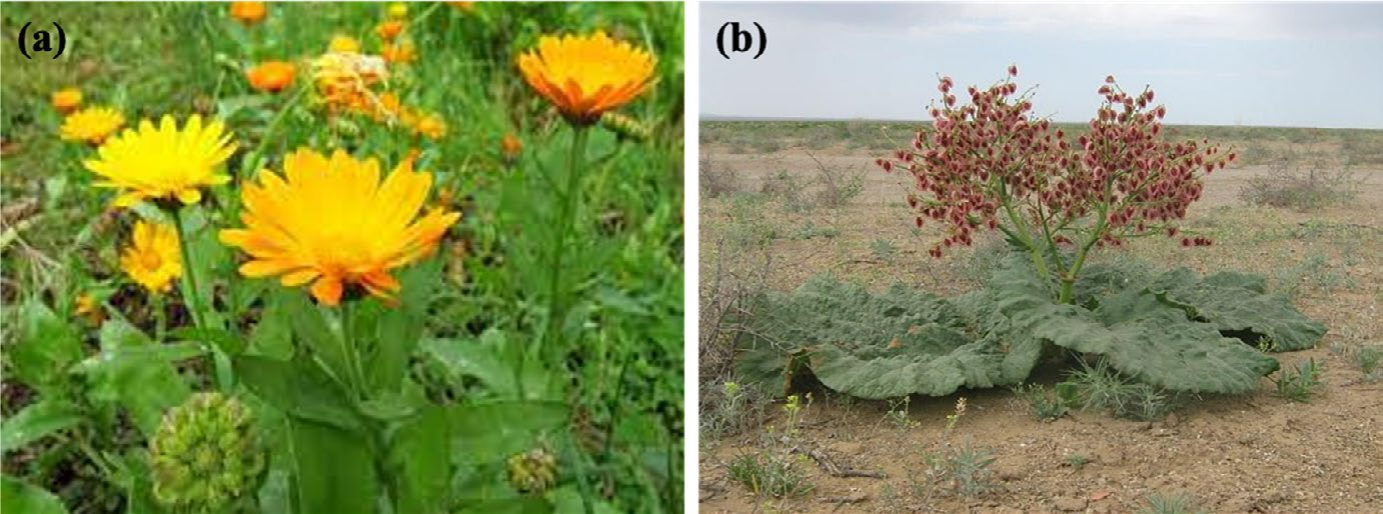
The pictures of (a) *Calendula officinalis* flowers and (b) *Rheum turkestanicum*.

*R. turkestanicum* was collected from Southwest Kophetdag (37.881352, 58.539756) in Turkmenistan. *C. officinalis* flowers were collected from the vicinity of Khivaabad (37.195390, 59.549774) in Kaka, Akhal district. The plant diagnosis was confirmed by The National Institute of Deserts, Flora and Fauna of Turkmenistan (Figure 1).

### 2.2 Preparation of COF and RT Extracts

The flowers of these plants were air‐dried at room temperature. Subsequently, the dried plant materials were finely ground using a laboratory mill. Two plant specimens were subjected to dehydration in an oven at 58°C for a duration of 24 h. We have selected Soxhlet for ethanol extraction. This extraction condenses and recycles the solvent upon contact with the material, maintaining the concentration gradient between the interior and exterior of the material, so facilitating dynamic extraction. As for the infusion technique, it has traditionally been used for the preparation of most plants. For the aqueous extract, 10 g of powdered substance from two specimens were infused in 200 mL of distilled water for 15 min. Afterward, the mixture was filtered through a Whatman No.1 filter and then subjected to lyophilization. Secondly, a total of 15 g of the powdered plant material was combined with 250 mL of ethanol and subjected to extraction in a Soxhlet apparatus for 6–8 h. The resulting extracts were concentrated under vacuum at 40°C using a rotary evaporator. The aqueous extracts were then filtered and subjected to lyophilization at −80°C for 48 h. The obtained extracts were stored at +4°C in the dark until ready for use.

### 2.3 Measurement of Total Bioactive Contents

As previously described, the Folin–Ciocalteu (FC) reagent method was employed to assess the total bioactive content in various extracts of COF and RT (Tousif et al. 2022; Zubair et al. 2022). The total phenolic content (TPC) was determined by measuring the amount of gallic acid equivalent (mg GAE/g) per gram of extract. Similarly, the total flavonoid content (TFC) was calculated using the rutin equivalent (mg RE/g extract) per gram of extract (Saleem et al. 2021; Shazmeen et al. 2022).

### 2.4 UHPLC–MS/MS Analysis of Polyphenolic Compounds

UHPLC–MS/MS analysis was performed using a Dionex Ultimate 3000 UHPLC system equipped with TSQ Quantum Access Max triple‐quadrupole mass spectrometer (ThermoFisher Scientific, Basel, Switzerland). The elution was performed at 40°C on a Syncronis C18 column (100 × 2.1 mm, 1.7 μm particle size). All chromatographic and MS quantification parameters were previously described in Radović et al. (2020). Xcalibur software version 2.2 (Thermo Fisher Scientific, San Jose, CA, USA) was used for instrument control, data acquisition, and data analysis. The phenolics were identified by direct comparison with commercial standards and expressed as mg/kg (Korpayev et al. 2023).

### 2.5 Antioxidant Assays

Through the use of reducing power (FRAP and CUPRAC), radical scavenging (DPPH and ABTS), and the phosphomolybdenum assay, the antioxidant capability of each extract was evaluated. The results were reported as trolox equivalents (mg TE/g extract). While the results of the metal chelating power assay, which was also used to determine antioxidant activity, were expressed as mg EDTAE/g extract (Tousif et al. 2022).

### 2.6 Enzyme Inhibition Studies

The ability of each extract to inhibit the enzymes acetylcholinesterase (AChE), butyrylcholinesterase (BChE), tyrosinase, ‐amylase, and ‐glucosidase was determined using in vitro standard methods as previously reported (Luisi et al. 2018; Saleem et al. 2021; Shazmeen et al. 2022).

### 2.7 Evaluation of the Antimicrobial Activity

Antibacterial and antifungal activities were determined using the microdilution method previously described (Zengin et al. 2017). The microorganisms were obtained from the Department of Plant Physiology, Institute for Biological Research “Siniša Stanković,” University of Belgrade, National Institute of the Republic of Serbia.

#### 2.7.1 Cytotoxicity Toward HaCaT Cells

The cytotoxic effect of the extracts was assessed on the spontaneously immortalized keratinocyte cell line (HaCaT) using a crystal violet assay, following a previously described method with some modifications (Stojković et al. 2020). The criterion used to categorize the cytotoxicity of preparations in the HaCaT cell line was as follows: IC_50_ ≤ 20 μg/mL = highly cytotoxic, IC_50_ ranged between 21 and 200 μg/mL = moderately cytotoxic, IC_50_ ranged between 201 and 400 μg/mL = weakly cytotoxic, and IC_50_ > 401 μg/mL = no cytotoxicity.

### 2.8 Molecular Docking

The proteins and enzymes utilized in this study were obtained from the Protein Data Bank (PDB), with detailed information provided in Table S1 for reference. The co‐crystallized ligands, cofactors, and water molecules were removed using BIOVIA Discovery Studio Visualizer V4.5. The ligands were obtained from PubChem and subsequently optimized using OpenBabel V3.1.1. The protein and enzyme structures were prepared using MGL Tools, version 1.5.6. The active sites within the proteins and enzymes were identified using POCASA V1.1, inhibitor binding sites, or methods supported by the literature (Table S1) (Duran et al. 2024; Yu et al. 2010). To validate the docking results, a re‐docking process was conducted. The ligand was re‐docked with the protein, and the root mean square deviation (RMSD) values were calculated to assess the accuracy of the docking. The RMSD, which measures the average deviation between the positions of atoms in the reference and target structures, was calculated using the following formula:

$$\text{RMSD}=\sqrt{\frac{1}{N}\sum_{i=1}^{N}{\left(r_{i}^{\mathrm{ref}}-r_{i}^{\text{target}}\right)}^{2}}$$

Molecular docking was performed using AutoDock Vina V1.1.2, with grid boxes set according to the methodology described by Trott and Olson (2010).

### 2.9 MM/PBSA Binding Free Energy Calculation

In this study, the *gmx_MMPBSA* tool (https://valdes‐tresanco‐ms.github.io/gmx_MMPBSA/dev/getting‐started/) was used to evaluate the stability of the molecules and to perform free energy calculations. In this study, 5‐ns molecular dynamics (MD) simulations were conducted, and the most stable molecules were selected based on the obtained data. These selected molecules were then subjected to longer MD simulations (100 ns) (Miller III et al. 2012; Valdés‐Tresanco et al. 2021).

### 2.10 MD Simulation

MD simulations were initiated using the CHARMM graphical user interface (GUI) platform (https://charmm‐gui.org/) and configured using the Solution Builder tool (Jo et al. 2008). The proteins were parameterized using the CHARMM36m force field (Maier et al. 2015; Yagi et al. 2024). The simulation system was enclosed in a periodic boundary box filled with TIP3P water molecules, ensuring a minimum distance of 10 Å between the protein and the box edges. To neutralize the system, counterions were added to bring the NaCl concentration to 0.15 M. Electrostatic and van der Waals interactions were treated using the Verlet cutoff scheme, while bond lengths were constrained using the LINCS algorithm. Long‐range electrostatics were calculated using the particle mesh Ewald (PME) method. Energy minimization was performed using the steepest descent algorithm until potential energy changes were reduced below 1000 kJ/mol/nm. The system was then equilibrated through NVT and NPT phases at 300 K to ensure thermodynamic stability. The fabrication simulation was run for 100 ns (nstep = 50,000,000) using GROMACS 2023.1. Post‐simulation trajectory analysis was performed using the gmx_energy, gmx_rms, gmx_rmsf, and gmx_gyrate modules to evaluate parameters such as total energy, RMSD, RMSF, hydrogen bond count, solvent accessibility, and gyration radius.

### 2.11 Statistical Analysis

All tests were conducted in triplicate, and the results are presented as mean values with standard deviation (SD) using Microsoft Excel. To differentiate between samples, hierarchical cluster analysis (HCA) plots were constructed in Morpheus software (Broad Institute 2024), based on the Spearman method of cluster agglomeration, adopting the average linkage method.

## 3 Results and Discussion

### 3.1 Total Phenolic and Flavonoid Contents

Based on Table 1, the infusion of COF has a higher concentration of TPC compared to ethanol, with values of 40.99 ± 0.87 mg GAE/g for infusion and 13.35 ± 0.23 mg GAE/g for ethanol. On the other hand, the TFC of COF remains relatively constant between the infusion and ethanol extraction methods, with values of 13.47 ± 0.51 mg RE/g for infusion and 13.43 ± 0.1 mg RE/g for ethanol. When comparing the TPC and TFC values of COF obtained by infusion and ethanol extraction, it can be observed that the TPC value is significantly higher in the infusion method compared to the ethanol extraction method. This could be attributed to the fact that phenolic compounds are generally more soluble in water and are thus more readily extracted by the infusion method, which involves steeping the plant material in hot water. This observation aligns with literature findings, which report higher solubility of phenolic compounds in water, facilitating their extraction through infusion (Dey and Kuhad 2014; Hossain et al. 2011). However, the TFC values remain relatively constant between the two extraction methods, which may indicate that flavonoids are extracted equally well by both methods. For RT, the ethanol extract has a higher concentration of TPC compared to the infusion method, with a value of 75.82 ± 0.18 mg GAE/g for ethanol and 45 ± 0.6 mg GAE/g for infusion. This trend is consistent with existing research, which suggests that certain phenolic compounds exhibit greater solubility in ethanol than in water, leading to higher TPC values in ethanol extracts (Ainsworth and Gillespie 2007; Zhang, Yang, and Wang 2011). In contrast, for RT, the ethanol extraction method resulted in a significantly higher TPC value compared to the infusion method. This may be due to the fact that some phenolic compounds are more soluble in ethanol than in water. However, since the TFC value was not detected in the infusion method, it is difficult to compare the flavonoid content between the two methods for this particular species. It is worth noting that the values reported in this table are specific to the methods used for extraction and the sample tested. Different extraction methods or different parts of the same plant may yield different results in terms of TPC and TFC. For comparing these two different species: when comparing the TPC and TFC values of COF and RT, it is observed that the TPC values are higher for RT in both extraction methods. This may be due to differences in the phenolic composition of the two plant species, as well as the fact that different extraction methods may preferentially extract certain types of phenolic compounds. The TFC values, on the other hand, are only reported for COF, and thus a direct comparison with RT cannot be made. However, it is worth noting that flavonoids are a diverse group of compounds that can vary greatly in structure and solubility, and thus the flavonoid content can vary widely between plant species (Manach et al. 2004).

**TABLE 1:** Total phenolic, flavonoid infusion, and ethanol extract of COF and RT.

Samples	Extracts	TPC (mg GAE/g)	TFC (mg RE/g)
COF	Infusion	40.99 ± 0.87	13.47 ± 0.51
	Ethanol	13.35 ± 0.23	13.43 ± 0.1
RT	Infusion	45.00 ± 0.6	Nd
	Ethanol	75.82 ± 0.18	Nd

*Note:* Values expressed are means ± SD of three parallel measurements.

Abbreviations: GAE, gallic acid equivalent; Nd, not determined; RE, rutin equivalent; TPC, total phenolic content; TFC, total flavonoid content.

### 3.2 UHPLC–MS/MS Quantitative Analysis

Table 2 lists concentrations of various compounds found in COF and RT (aqueous and ethanol extracts). The compounds and their concentrations vary between the different species and extracts. HCA was performed on the quantitative data, where the content of the tested compounds in the given extracts was illustrated (Figure S1). For example, ethanol extract of COFs has higher concentrations of most compounds compared to aqueous extract of COF and RT. Some compounds are present in only one or two of the species, while others are present in all four extracts. There is variation in the concentrations of individual compounds even within the same species and type of extract. For example, the concentration of 5‐O‐caffeoylquinic acid is much higher in COF (ethanol) compared to COF (aqueous), while the concentration of *p*‐coumaric acid is much higher in COF (aqueous) compared to COF (ethanol). This pattern of variability is in line with other research showing that the extraction efficiency of some phenolic compounds can be significantly impacted by the solubility of those compounds in different solvents (Chemat, Vian, and Cravotto 2012; Ignat, Volf, and Popa 2011).

**TABLE 2:** Contents (μg/g of dry weight extract) of bioactive compounds in COF and RT infusion and ethanol extracts. The analysis was performed in triplicate.

Species, mg/kg	COF (infusion)	COF (ethanol)	RT (infusion)	RT (ethanol)
3‐O‐Caffeoylquinic acid	82.91 ± 1.40	749.29 ± 1.88	1.07 ± 0.04	0.17 ± 0.01
5‐O‐Caffeoylquinic acid	378.40 ± 15.27	2780.56 ± 47.80	11.37 ± 0.71	109.34 ± 8.40
Caffeic acid	22.00 ± 0.13	86.13 ± 2.00	0.89 ± 0.03	NF
Isoorientin	NF	1.23 ± 0.08	NF	NF
Rutin	213.44 ± 4.06	1356.97 ± 60.30	5.03 ± 0.15	1.83 ± 0.09
Vitexin	NF	0.95 ± 0.06	1.49 ± 0.02	4.81 ± 0.18
*p*‐Coumaric acid	77.84 ± 1.71	25.02 ± 1.47	6.35 ± 0.08	NF
Quercetin 3‐O‐glucoside	86.83 ± 6.28	247.72 ± 7.69	8.04 ± 0.07	8.10 ± 0.00
Catechin gallate	NF	NF	21.66 ± 0.62	80.01 ± 2.59
Isorhamnetin 3‐O‐rutinoside	460.54 ± 20.76	1653.59 ± 117.93	NF	NF
Isorhamnetin 3‐O‐glucoside	171.29 ± 3.19	336.80 ± 16.02	NF	NF
Quercetin 3‐O‐rhamnoside	NF	NF	NF	2.54 ± 0.27
Kaempferol 3‐O‐glucoside	9.44 ± 0.12	25.68 ± 0.22	NF	NF
Eriodictyol	NF	2.65 ± 0.12	0.43 ± 0.00	NF
Quercetin	33.96 ± 1.65	11.64 ± 0.41	0.98 ± 0.02	NF
Naringenin	NF	NF	0.99 ± 0.07	1.04 ± 0.06
Apigenin	NF	NF	6.53 ± 0.22	NF
Kaempferol	14.75 ± 0.66	8.30 ± 0.36	NF	372.61 ± 2.63
Hispidulin	0.24 ± 0.03	0.55 ± 0.04	NF	NF
Isorhamnetin	806.96 ± 43.31	212.18 ± 9.24	NF	NF

Abbreviation: NF, not found.

COF (aqueous) has higher concentrations of quercetin, 3‐O‐caffeoylquinic acid, and rutin compared to COF (ethanol). COF (ethanol) has higher concentrations of most other compounds, including 5‐O‐caffeoylquinic acid, caffeic acid, and vitexin. COF (ethanol) has higher concentrations of most other compounds, including 5‐O‐caffeoylquinic acid, caffeic acid, and vitexin. COF (aqueous) and COF (ethanol) have similar compounds present, but COF (ethanol) generally has higher concentrations of those compounds. This might be attributed to the broader solvent capabilities of ethanol, which can dissolve a wider range of phytochemicals compared to water (Handa et al. 2008). In addition, several writers have reported in the literature on the varying levels of individual and total chemicals found in the various sections of *C. officinalis* (Bekdeşer 2019; Ourabia et al. 2019).

To summarize, Table 2 provides a starting point for understanding the chemical composition of these plant species and the variation that exists within and between them. However, more context and analysis would be needed to draw any meaningful conclusions or make comparisons between the different species and extracts.

### 3.3 Antioxidant Effects

The table shows the antioxidant activity of COF and RT extracts, as determined by several methods including PBD, DPPH, ABTS, CUPRAC, FRAP, and MCA (Table 3). In general, it can be observed that the ethanol extracts of both plant species exhibit higher antioxidant activity compared to the infusion extracts for most of the tested methods. This could be attributed to the fact that ethanol is a stronger solvent than water and can thus extract more phenolic compounds with antioxidant activity (Garmus et al. 2014).

**TABLE 3:** Antioxidant properties of the tested extracts.

Samples	Extracts	PBD (mmol TE/g)	DPPH (mg TE/g)	ABTS (mg TE/g)	CUPRAC (mg TE/g)	FRAP (mg TE/g)	MCA (mg EDTAE/g)
COF	Infusion	0.95 ± 0.01	13.2 ± 1.8	101.36 ± 0.48	113.22 ± 0.71	67 ± 0.6	13.4 ± 0.83
	Ethanol	1.11 ± 0.24	9.34 ± 0.14	21.96 ± 0.1	49.07 ± 0.77	24.25 ± 0.34	4.12 ± 0.49
RT	Infusion	0.86 ± 0.03	45.51 ± 0.33	111.61 ± 0.06	143.2 ± 0.55	84.54 ± 0.5	27.09 ± 0.11
	Ethanol	1.52 ± 0.11	171.5 ± 0.13	382.35 ± 2.95	449.80 ± 1.72	195.6 ± 4.4	5.84 ± 0.17

*Note:* Values expressed are means ± SD of three parallel measurements.

Abbreviations: EDTAE, EDTA equivalent; MCA, metal chelating assay; PBD, phosphomolybdenum assay; TE, trolox equivalent.

For COF, both the ethanol and infusion extracts exhibited similar levels of total antioxidant activity, as measured by the PBD assay. However, the ethanol extract had higher activity than the infusion extract for all the other assays (DPPH, ABTS, CUPRAC, FRAP, and MCA). This suggests that the ethanol extract of COF has higher levels of phenolic compounds with antioxidant activity than the infusion extract (Do et al. 2014).

For COF, the highest antioxidant activity was observed for the CUPRAC method in both extraction methods. The FRAP method also showed relatively high antioxidant activity, while the DPPH and ABTS methods showed lower activity. The MCA method showed the lowest antioxidant activity for both extraction methods. For RT, the highest antioxidant activity was observed for the ethanol extracts using the ABTS and CUPRAC methods, while the FRAP method showed the lowest activity. The MCA method also showed relatively low antioxidant activity for both extraction methods. Comparing the antioxidant activity between COF and RT, it can be observed that the ethanol extract of RT had higher antioxidant activity than the ethanol extract of COF for all the tested assays (PBD, DPPH, ABTS, CUPRAC, FRAP, and MCA). This suggests that *Rheum tanguticum* has higher levels of phenolic compounds with antioxidant activity than COF, which aligns with literature reports that different species possess varying levels of bioactive compounds (Prior, Wu, and Schaich 2005). However, for the infusion extracts, COF had higher levels of antioxidant activity than RT for all the assays except for the ABTS assay. This suggests that the infusion extract of COF has higher levels of phenolic compounds with antioxidant activity than the infusion extract of RT. Antioxidant qualities of the hydroalcoholic extract from COF were previously reported by Ak et al. (2020). It is important to note that different antioxidant assays measure different aspects of antioxidant activity and may be influenced by various factors such as pH, solvent, temperature, and the presence of other compounds in the sample (Kim, Seong, and Chung 2020; Munteanu and Apetrei 2021). Therefore, it is recommended to use multiple assays to evaluate the antioxidant activity of a sample and to interpret the results with caution (Huang, Ou, and Prior 2005).

### 3.4 Enzyme Inhibitory Effects

Our research focused on evaluating the extracts for anti‐cholinesterase, anti‐tyrosinase, anti‐amylase, and anti‐glucosidase activities. We focused primarily on these enzymes because of their critical role in addressing global health challenges. Cholinesterase inhibitors are crucial for relieving the symptoms of Alzheimer's disease (Chen et al. 2023). Amylase and glucosidase inhibitors are important to alleviate the effects of diabetes mellitus in patients following a high‐carbohydrate diet (Visvanathan et al. 2024). Additionally, inhibiting tyrosinase is crucial for treating hyperpigmentation problems caused by excessive melanin production (Nisa et al. 2024). Based on this information, we selected the enzyme to evaluate the potential of the tested extracts for global health problems.

The following Table 4 presents the results of various bioactivity assays conducted on COF and RT extracts, including their AChE, BChE, α‐amylase, α‐glucosidase, and tyrosinase inhibition activities. The ethanol extract showed a significantly higher inhibitory effect on all enzymes tested compared to the infusion extract. For example, the AChE inhibitory activity of the ethanol extract was 2.97 ± 0.0053 mg GALAE/g, while that of the infusion extract was 2.02 ± 0.11 mg GALAE/g. Similarly, the tyrosinase inhibitory activity of the ethanol extract was 71.74 ± 0.17 mg KAE/g, while that of the infusion extract was 7.73 ± 1.28 mg KAE/g. However, it is worth noting that the BChE inhibitory activity was only detectable in the ethanol extract. These results are consistent with findings in the literature where ethanol extracts of various plant species often demonstrate superior bioactivity compared to aqueous extracts due to the higher solubility of bioactive compounds in ethanol (Plaskova and Mlcek 2023; Sepahpour et al. 2018). Furthermore, unlike drawn‐out aqueous extractions that could break down bioactive components, ethanol extraction not only permits improved solubilization but also maintains thermolabile chemicals throughout processing (Lezoul et al. 2020; Lohvina, Sándor, and Wink 2022).

**TABLE 4:** Enzyme inhibitory effects of the infusion and ethanol extracts of COF and RT.

Samples	Extracts	AChE (mg GALAE/g)	BChE (mg GALAE/g)	Amylase (mmol ACAE/g)	Glucosidase (mmol ACAE/g)	Tyrosinase (mg KAE/g)
COF	Infusion	2.02 ± 0.11	Na	0.09 ± 0.001	0.09 ± 0.01	7.73 ± 1.28
	Ethanol	2.97 ± 0.0053	2.9 ± 0.08	0.29 ± 0.012	0.96 ± 0.01	71.74 ± 0.17
RT	Infusion	1.24 ± 0.022	0.04 ± 0.018	0.06 ± 0.01	0.96 ± 0.01	8.87 ± 0.33
	Ethanol	3.0 ± 0.011	2.86 ± 0.044	0.32 ± 0.01	1.01 ± 0.02	72.43 ± 0.39

*Note:* Values expressed are means ± SD of three parallel measurements.

Abbreviations: ACEs, acarbose equivalents; GALAEs, galantamine equivalents; KAEs, kojic acid equivalents.

RT aqueous extract showed comparable inhibitory activity to ethanol extract on AChE, BChE, and α‐glucosidase, but had a significantly higher inhibitory activity on α‐amylase and tyrosinase. According to reports, there is an α‐amylase inhibitory activity in the ethanolic extract of *C. officinalis* leaves (Olennikov and Kashchenko 2014). To illustrate, the tyrosinase inhibitory activity of RT ethanol extract was 72.43 ± 0.39 mg KAE/g, while that of COF ethanol extract was 71.74 ± 0.17 mg KAE/g. These findings are consistent with earlier studies on Rheum species, which have been emphasized for their phenolic‐rich profiles comprising stilbenoids and anthraquinones, which are known to block enzymes that hydrolyze carbohydrates, including α‐amylase and α‐glucosidase (Dehghan, Salehi, and Amiri 2018). Although specific component analysis would be required to verify these hypotheses, the comparable tyrosinase inhibition between RT and COF extracts further suggests that both plants may contain structurally similar phenolics. Although specific component analysis would be required to verify these hypotheses, the comparable tyrosinase inhibition between RT and COF extracts further suggests that both plants may contain structurally similar phenolics. RT infusion extract had a significantly lower inhibitory activity on AChE and a significantly higher inhibitory activity on α‐amylase and tyrosinase compared to COF infusion extract. For example, the tyrosinase inhibitory activity of RT infusion extract was 8.87 ± 0.33 mg KAE/g, while that of Calendula infusion extract was 7.73 ± 1.28 mg KAE/g. However, BChE inhibitory activity was only detected in COF infusion extract. Interestingly, BChE inhibitory activity was only detected in COF infusion extract, consistent with literature showing that some plants exhibit selective enzyme inhibition based on the extraction method used (Silva et al. 2021). When specifically inhibiting specific enzymes for pharmaceutical or nutraceutical purposes, this heterogeneity highlights the importance of optimizing extraction techniques according to the targeted bioactivities (Quitério et al. 2022).

### 3.5 Antibacterial Activity

In this study, the antibacterial susceptibility of extracts COF and RT as infusion and ethanol for both species is demonstrated in Table 5. The results of the bacteriostatic (MIC) and bactericidal (MBC) effects of all extracts compared to that of two reference drugs, streptomycin and ampicillin. Table 5 is divided into two parts, COF (infusion) and COF (ethanol), and RT (infusion) and RT (ethanol). The MIC and MBC of the COF (infusion) against the bacterial strains is higher than in part COF (ethanol). The best activity of COF (ethanol) is presented on *Bacillus cereus* with 0.188 mg/mL (MIC) and 0.250 mg/mL (MBC). In part RT (infusion), the MIC and MBC of the extract against all the bacterial strains are relatively lower than RT (ethanol). Antibacterial activity exists on most tested species with the exception of RT infusion and ethanol on *Micrococcus lutes*. This table provides valuable information on the susceptibility of extracts to different bacterial strains, which can help in the selection of appropriate extracts with proper solvents for the treatment of bacterial infections. Comparing the values between conventional antibiotics and extracts, it can be seen that in general, the RT (infusion) and RT (ethanol) have lower MIC and MBC values against *Staphylococcus aureus* , *Listeria monocytogenes*, *Escherichia coli* , *Salmonella typhimurium* , and *Enterobacter* *cloacae*, indicating greater antibacterial activity. For example, against *S. aureus* and *L. monocytogenes* , the MIC/MBC of RT (infusion) (0.06–0.125 mg/mL) is much lower than that of conventional antibiotics (0.1–0.2 mg/mL). This finding aligns with reports that certain plant extracts exhibit potent antibacterial activity and could serve as alternatives to conventional antibiotics (Prasad, Zolnik, and Molina 2019). Similarly, for *B. cereus* , the MIC/MBC of COF (ethanol) (0.188–0.250 mg/mL) is similar to that of conventional antibiotics (0.1–0.2 mg/mL). However, there are a few exceptions to this trend. Against *Pseudomonas aeruginosa* , the MIC and MBC values for extracts are much higher (2.0–4.0 mg/mL) than those for conventional antibiotics (0.1–0.2 mg/mL). This trend is also observed for *L. monocytogenes* , *Micrococcus luteus* , and *P. aeruginosa* . Against *E. coli* , the MIC and MBC values for extracts are higher (0.125–2.0 mg/mL) than those for conventional antibiotics (0.1–0.2 mg/mL) (Table 5). This variability highlights the importance of selecting appropriate extraction methods and solvents to optimize antibacterial activity (Casagrande et al. 2018; Jovanović et al. 2021). The search for new antimicrobial molecules is today urgent due to the diffusion of infecting agents and resistant forms of microorganisms. Thus, the natural source of compounds with potential activity in this regard can be a valuable source of new drugs, for this reason extensive research into plant antimicrobials is today needed both considering purified compounds as well as plant extracts (Stojković et al. 2023). To summarize, Table 5 suggests that extracts have promising antibacterial activity against a range of bacterial strains, and further research into their potential use as alternative or complementary therapies for bacterial infections is warranted.

**TABLE 5:** Antibacterial activity of *Calendula officinalis* and *Rheum turkestanicum* extracts (mg/mL).

		*S.a*.	*B.c*.	*L.m*.	*M.l*.	*P.ae*.	*E.c*.	*S.t*.	*En.cl*.
COF (infusion)	MIC	4.0	1.5	3.0	3.0	1.5	3.0	3.0	3.0
	MBC	8.0	2.0	4.0	4.0	2.0	4.0	4.0	4.0
COF (ethanol)	MIC	1.5	0.188	1.5	1.5	1.0	1.5	1.0	1.5
	MBC	2.0	0.250	2.0	2.0	2.0	2.0	2.0	2.0
RT (infusion)	MIC	0.06	0.5	0.06	≥ 8.0	2.0	0.125	0.125	0.25
	MBC	0.125	1.0	0.125	≥ 8.0	4.0	0.25	0.25	0.5
RT (ethanol)	MIC	0.125	0.250	0.125	≥ 8.0	3.0	1.0	0.125	1.0
	MBC	0.250	0.5	0.250	≥ 8.0	4.0	2.0	0.250	2.0
Streptomycin	MIC	0.100	0.025	0.150	0.050	0.100	0.100	0.100	0.025
	MBC	0.200	0.050	0.300	0.100	0.200	0.200	0.200	0.050
Ampicillin	MIC	0.100	0.100	0.150	0.100	0.300	0.150	0.100	0.100
	MBC	0.150	0.150	0.500	0.150	0.500	0.200	0.200	0.150

*Note:* Minimum inhibitory concentration (MIC) and minimum bactericidal concentration (MBC) values.

Abbreviations: S.a., *Staphylococcus aureus* ; B.c., *Bacillus cereus* ; *L.m*, *Listeria monocytogenes* ; M.l., *Micrococcus luteus* ; P.a., *Pseudomonas aeruginosa* ; E.c., *Escherichia coli* ; S.t., *Salmonella typhimurium* ; En.cl, *Enterobacter cloacae* .

### 3.6 Antifungal Activity

Table 6 shows the results of susceptibility testing for several fungal strains against different extracts COF and *Rheum turkestanicum* (RT) as antifungal agents. The first column lists the extract tested, followed by the minimum inhibitory concentration (MIC) and minimum fungicidal concentration (MFC) values for each strain. The best results in terms of MIC/MFC were observed regarding *Penicillium funiculosum*. In depth *P. funiculosum* was inhibited/killed by COF ethanol extract at 0.375 and 0.5 mg/mL respectively These concentrations were significantly lower in relation to all other tested extracts. Moreover, the MFC value was the same as the value of the ketoconazole (0.5 mg/mL). In terms of MIC values, *Aspregillus fumigatus*, *Aspregillus versicolor*, and *Aspregillus flavus* were inhibited by all antifungal agents tested, whereas *Aspregillus niger* had a MIC value of ≥ 8.0 for all extracts. *Trichoderma viride* had a similar MIC value for most extracts compared to the other species (1–1.5 mg/mL). *P. funiculosum*, *Penicillium ochrochloron*, and *Penicillium verrucosum* var. *cyclopium* had varying MIC values for each agent, indicating some variability in their sensitivity to the extracts tested. When comparing MFC values of extracts, *A. fumigatus* and *A. versicolor* had MFC values ≤ 4.0 for all agents. This is consistent with studies that demonstrate the effective fungicidal activity of certain plant extracts at low concentrations (Sales et al. 2016). *A. niger* had an MFC value of ≥ 8.0 for all extracts, indicating resistance to the extracts tested. *T. viride* had promising MFC values for most agents compared to the other species, indicating reduced susceptibility to the extracts. *P. funiculosum*, *P. ochrochloron*, and *P. verrucosum* var. *cyclopium* had varying MFC values for each extract, indicating variability in antifungal extracts' susceptibility to the strains (Lopes et al. 2018), a trend supported by research highlighting variable effectiveness of antifungal agents across different fungi (Chiavaroli et al. 2020).

**TABLE 6:** Antifungal activity of *Calendula officinalis* and *Rheum turkestanicum* extracts (mg/mL).

		*A.f*.	*A.n*.	*A.v*.	*A.fl*.	*T.v*.	*P.f*.	*P.o*.	*P.v.c*.
COF (infusion)	MIC	2.0	≥ 8.0	2.0	2.0	1.0	1.0	2.0	1.5
	MFC	4.0	≥ 8.0	4.0	4.0	2.0	2.0	4.0	2.0
COF (ethanol)	MIC	2.0	≥ 8.0	2.0	2.0	1.0	0.375	2.0	2.0
	MFC	4.0	≥ 8.0	4.0	4.0	2.0	0.5	4.0	4.0
RT (infusion)	MIC	2.0	4.0	2.0	2.0	1.5	3.0	2.0	3.0
	MFC	4.0	8.0	8.0	4.0	4.0	4.0	4.0	4.0
RT (ethanol)	MIC	2.0	8.0	4.0	2.0	1.0	6.0	1.0	8.0
	MFC	4.0	≥ 8.0	8.0	4.0	2.0	8.0	2.0	≥ 8.0
Bifonazole	MIC	0.150	0.150	0.100	0.150	0.150	0.200	0.200	0.100
	MFC	0.200	0.200	0.200	0.200	0.200	0.250	0.250	0.200
Ketoconazole	MIC	0.200	0.200	0.200	0.200	1.000	0.200	1.000	0.200
	MFC	0.500	0.500	0.500	0.500	1.500	0.500	1.500	0.300

*Note:* Minimum inhibitory concentration (MIC) and minimum fungicidal concentration (MFC) values.

Abbreviations: A.f., *Aspregillus fumigatus*; A.n., *Aspergillus niger*; A.v., *Aspergillus versicolor*; A.fl., *Aspergillus flavus*; T.v, *Trichoderma viride*; P.f., *Penicillium funiculosum*; P.o., *Penicillium ochrochloron*; P.v.c., *Penicillium verrucosum* var. *cyclopium*.

From the results, it can be seen that the antibacterial activity of the tested extracts is better than the antifungal activity. The extracts derived from two different parts and solvents looked chemically and biologically dissimilar. The knowledge gained in the current work showed that the solvent of the extraction and the plant parts emerged as the main factors in chemical and biological activity. In search of new antifungal agents (Ivanov, Ćirić, and Stojković 2022) effective to fight against more and more resistant microfungi it is crucial to discover new sources from plants.

### 3.7 Cytotoxic Results

The evaluation of drug cytotoxicity is an important step in biomedical research and represents a primary consideration covering drug selection. Additionally, the first step in the development of novel antimicrobial drugs includes toxicity studies on human cells in culture. The cytotoxic effect of the COF and RT extracts was assessed on the HaCaT cell line, a spontaneously transformed aneuploid immortal keratinocyte cell line from adult human skin, a very sensible cell line used as an effective in vitro alternative for an initial orientating screening of safety issues of substances. All tested extracts expressed no cytotoxicity toward this cell line with IC_50_ value > 400 μg/mL (results not presented in the table since all 4 extracts possessed no cytotoxicity up to 400 μg/mL, higher IC_50_ value is considered non‐toxic to human cells). In a previous study (Martins de Deus et al. 2023), a hydroethanolic extract of COF was tested on various cell lines, including AGS (human gastric adenocarcinoma), CaCo‐2 (human colon adenocarcinoma), MCF‐7 (human breast adenocarcinoma), VERO (kidney epithelial cells from African green monkey), and PLP2 (porcine liver primary cell culture). The GI_50_ values ranged from 214 to 360 μg/mL, which aligns with moderate cytotoxicity depending on the cell type. Generally, a GI_50_ value within this range indicates a tolerable cytotoxic profile, as it is close to or above the 200 μg/mL threshold, often considered as a benchmark for weakly‐toxic effects in human cell lines. These results align with our study, indicating weak cytotoxicity; however, differences in concentration values might be attributed to the polarity of the solvent used for hydro‐ethanolic extraction in the study by Martins de Deus et al. (2023). Shiezadeh et al. (2013) demonstrated that RT exhibits cytotoxic and apoptotic effects on HeLa and MCF‐7 cancer cell lines without inducing toxicity in normal cell lines, indicating a selective cytotoxic action favoring malignant over healthy cells. Furthermore, Moradzadeh et al. (2019) evaluated RT's cytotoxic effects on leukemic HL60 and NB4 cells, finding IC_50_ values of 518.60 and 597.80 μg/mL, respectively, after 24 h of treatment. Comparing these results to findings in HaCaT cells, our study observed no significant cytotoxicity from RT extracts in this human keratinocyte line. RT's lack of cytotoxicity in HaCaT cells supports its selectivity, reinforcing the idea that it preferentially targets cancerous cells while sparing both normal and skin‐derived cells. This selective cytotoxic profile is a promising indication that RT may have therapeutic potential for treating malignancies without damaging non‐cancerous cells, especially those in skin applications where HaCaT cell compatibility is relevant. Our findings add to the body of research by showing that COF and RT extracts are safe for skin cells specifically, indicating potential for topical applications or therapeutic uses where cytotoxicity is a concern.

### 3.8 Molecular Docking

**FIGURE 2:**
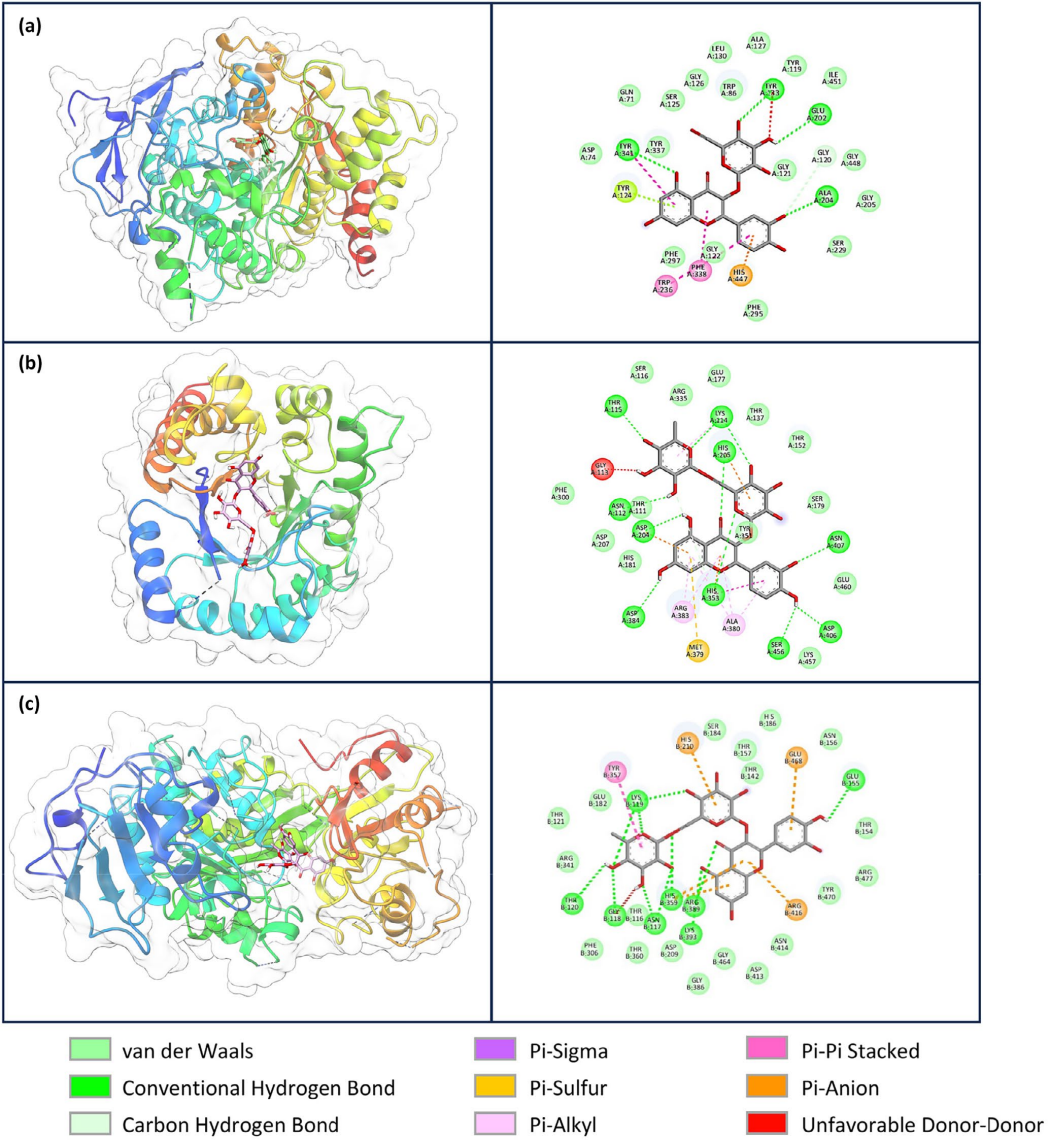
Enzymes and proteins' active sites with compounds showing the best binding energy. (a) Interaction between AChE and quercetin 3‐O‐glucoside. (b) Interaction between *S. aureus* dihydropteroate synthase and rutin. (c) Interaction between *E. coli* MurE and rutin.

The present study employed a comprehensive evaluation approach to assess the antimicrobial effects of hub molecules identified in *R. turkestanicum* and *C. officinalis* on specific bacterial enzymes, proteins, and standard enzymes. The coordinates and grid sizes necessary for these analyses are provided in Table S1. Among the multitude of compounds identified, isorhamnetin, p‐coumaric acid, quercetin 3‐O‐glucoside, 3‐O‐caffeoylquinic acid, 5‐O‐caffeoylquinic acid, isorhamnetin 3‐O‐rutinoside, rutin, and isorhamnetin 3‐O‐glucoside were selected for comprehensive analysis due to their widespread distribution. In the study, the selected proteins, including 30S ribosome S3, dihydropteroate synthase, gyrase B, MurE, and transpeptidase, were analyzed alongside the standard enzymes AChE, BChE, Tyr, amylase, and glucosidase, to examine the antimicrobial effects of hub molecules identified in *R. turkestanicum* and *C. officinalis* on these specific bacterial enzymes. Table 7 presents the compounds with binding energies lower than −8 kcal/mol, while Table S2 displays those with binding energies higher than −8 kcal/mol. The overall docking results demonstrated a range of binding energies, from −11.0 to −5.3 kcal/mol (Table S2). The lowest binding energies were observed with AChE and quercetin 3‐O‐glucoside (−11.0 kcal/mol) (Figure 2a), *S. aureus* dihydropteroate synthase and rutin (−10.9 kcal/mol) (Figure 2b), and *E. coli* MurE (−10.6 kcal/mol) (Figure 2c). In these interactions, non‐covalent interactions, including π–π stacking, π–σ, π–sulfur, π‐alkyl, and conventional hydrogen bonds, were observed to be more prevalent than hydrogen bonds.

**TABLE 7:** The docking score (kcal/mol) and interacting residues of the enzyme and protein.

Compound	Target	PDB ID	Binding energy	RMSD	Interaction		Binding site
					Type	Number	
Isorhamnetin	Amylase	2qv4	−9.0	0.16	H‐bond	3	Asp A:300, Tyr A:62, Gln A:63
Quercetin 3‐O‐glucoside	Amylase	2qv4	−8.9	1.10	H‐bond	2	Glu A:233, His A:299
3‐O‐Caffeoylquinic acid	Amylase	2qv4	−8.2	0.91	H‐bond	6	Arg A:195, His A:299, Asp A:300, Asp A:197, Glu A:233, Tyr A:62
5‐O‐Caffeoylquinic acid	Amylase	2qv4	−8.3	1.02	H‐bond	3	His A:305, His A:299, Asp A:197
Isorhamnetin 3‐O‐rutinoside	Amylase	2qv4	−9.6	1.08	H‐bond	4	Asp A:197, Glu A:233, Lys A:200, Gln A:63
Rutin	Amylase	2qv4	−9.4	0.86	H‐bond	6	His A:305 (3), Asp A:197, Glu A:233, Lys A:200
Isorhamnetin 3‐O‐glucoside	Amylase	2qv4	−8.4	1.05	H‐bond	2	Glu A:233, Gln A:63
Isorhamnetin	AChE	2y2v	−8.8	1.01	H‐bond	2	Trp A:86, Glu A:202
Quercetin 3‐O‐glucoside	AChE	2y2v	−11.0	1.05	H‐bond	4	Tyr A:133, Glu A:202, Ala A:204, Tyr A:341
3‐O‐Caffeoylquinic acid	AChE	2y2v	−9.3	1.01	H‐bond	2	Glu A:202, Tyr A:124
5‐O‐Caffeoylquinic acid	AChE	2y2v	−9.4	0.11	H‐bond	3	Tyr A:341 (2), Tyr A:124
Isorhamnetin 3‐O‐rutinoside	AChE	2y2v	−10.5	0.96	H‐bond	3	Leu A:76, Ser A:293, Tyr A:341
Rutin	AChE	2y2v	−10.1	1.07	H‐bond	4	Tyr A:337, Asp A:74, Ser A:293, Arg A:296
Isorhamnetin 3‐O‐glucoside	AChE	2y2v	−9.9	1.03	H‐bond	4	Glu A:202, Trp A:86 (2), Gly A:120
Isorhamnetin	BChE	3djy	−9.0	0.30	H‐bond	1	Glu A:197
Quercetin 3‐O‐glucoside	BChE	3djy	−10.1	0.98	H‐bond	8	Gly A:116 (2), Trp A:82, Gly A:115 (2), Tyr A:128, Asp A:70, Tyr A:332
3‐O‐Caffeoylquinic acid	BChE	3djy	−8.3	0.16	H‐bond	4	Pro A:285, Leu A:286, Ala A:199, Gly A:116
5‐O‐Caffeoylquinic acid	BChE	3djy	−8.3	5.32	H‐bond	4	Tyr A:332, Try A:128, Gly A:115, The A:120
Isorhamnetin 3‐O‐rutinoside	BChE	3djy	−11.0	0.80	H‐bond	6	Trp A:82 (2), Gly A:115, Gly A:116, Asp A:70, Pro A:285
Rutin	BChE	3djy	−10.8	0.66	H‐bond	4	Trp A:82, Tyr A:332, Ser A:287, Glu A:197
Isorhamnetin 3‐O‐glucoside	BChE	3djy	−10.1	0.63	H‐bond	4	Trp A:82, His A:438, Ala A:199, Leu A:286
Rutin	Tyr	5m8o	−9.0	0.20	H‐bond	5	Thr A:391, Asp A:212, The A:362, Asn A:378, Ser A:394
Isorhamnetin 3‐O‐rutinoside	*Dihydropteroate synthase*	1ad4	−8.2	0.42	H‐bond	2	Ser A:50, Arg A:219
Rutin	*Dihydropteroate synthase*	1ad4	−8.2	0.57	H‐bond	3	Arg A:66, Gly A:48, His A:55
Rutin	*Gyrase B*	4urn	−8.5	3.53	H‐bond	1	Ala A:122
Rutin	*30S ribosome S3*	5tcu	−8.0	1.07	H‐bond	8	Asp B:17 (2), Arg B:16, The B: 183, Asp B:43, Val B:54, Glu B:48, Arg A:113
Isorhamnetin	*MurE of S. aureus*	4c13	−9.3	8.53	H‐bond	3	Ser A:116, Thr A:115, His A:205
Quercetin 3‐O‐glucoside	*MurEof S. aureus*	4c13	−9.6	5.25	H‐bond	4	Thr A:111, His A:205, Thr A:351, Glu A:460
3‐O‐Caffeoylquinic acid	*MurE of S. aureus*	4c13	−9.8	0.04	H‐bond	11	Thr A:115, Lys A:114 (2), His A:205, Thr A:111, Asn A:301, Ser A:116, His A:353 (2), Gly A:113 (2)
5‐O‐Caffeoylquinic acid	*MurE of S. aureus*	4c13	−9.2	5.87	H‐bond	3	Asp A:350, Thr A:115, Thr A:152
Isorhamnetin 3‐O‐rutinoside	*MurE of S. aureus*	4c13	−10.5	2.22	H‐bond	7	Thr A:115, Gly A:113, Asn A:112, His A:353, Asp A:406, His A:205, Lys A:114
Rutin	*MurE of S. aureus*	4c13	−10.9	6.73	H‐bond	11	Thr A:115, Lys A:114 (2), His A:205, Asn A:407, Asp A:406, Ser A:456, His A:353, Asp A:384, Asp A:204, Asn A:212
Isorhamnetin 3‐O‐glucoside	*MurE of S. aureus*	4c13	−9.1	0.14	H‐bond	8	Ser A:456, Asp A:406, Arg A:383 (2), Ala A:150, Asn A:151, Thr A:115
Quercetin 3‐O‐glucoside	*Transpeptidase of S. aureus*	5tw8	−8.9	1.02	H‐bond	6	Ser A:139, Ser A:75, Ser A:116, Asn A:141, Glu A:114, Glu A:297
3‐O‐Caffeoylquinic acid	*Transpeptidase*	5tw8	−8.4	1.00	H‐bond	5	Ser A:75, Gly A:181, Asp A:264, Arg A:300, Glu A:297
5‐O‐Caffeoylquinic acid	*Transpeptidaseof S. aureus*	5tw8	−8.2	0.23	H‐bond	4	Thr A:260, Ser A:75 Asn A:141, Ser A:262
Isorhamnetin 3‐O‐rutinoside	*Transpeptidase of S. aureus*	5tw8	−9.1	0.11	H‐bond	5	Ser A:139, Ser A:75, Ser A:116, Asp A:264, Glu A:183
Rutin	*Transpeptidaseof S. aureus*	5tw8	−9.3	0.46	H‐bond	4	Thr A:260, Glu A:114, Ser A:75, Glu A:183
Isorhamnetin 3‐O‐glucoside	*Transpeptidaseof S. aureus*	5tw8	−8.7	0.94	H‐bond	8	Glu A:297, Thr A:260, Ser A:75, Ser A:139 (2), Ser A:116, Asn A:141, Glu A:114
Isorhamnetin	*MurE of E. coli*	1e8c	−8.6	0.18	H‐bond	4	Thr B:120, Asp B:356, Asn B:117, His B:359
Quercetin 3‐O‐glucoside	*MurE of E. coli*	1e8c	−9.5	0.04	H‐bond	5	Glu B:468, Lys B:393, Arg B:389, Glu B:182, His 210
3‐O‐Caffeoylquinic acid	*MurE of E. coli*	1e8c	−8.7	0.27	H‐bond	8	Thr B:120, Lys B:119, His B:359, Lys B:393, Asn B:416, Arg B:416, Glu A:468, Gly B:118
5‐O‐Caffeoylquinic acid	*MurE of E. coli*	1e8c	−8.6	0.66	H‐bond	8	Glu B:468, Arg B:416, Asn B:414, Thr B:120, Gly B:118, His B:359, Asp B:209, Lys B:293
Isorhamnetin 3‐O‐rutinoside	*MurE of E. coli*	1e8c	−9.6	0.18	H‐bond	6	Thr B:157, His B:467, Glu B:468, Gly B:464 (2), His B:359
Rutin	*MurE of E. coli*	1e8c	−10.6	1.08	H‐bond	11	Lys B:119 (3), Glu B:155, Arg B:389, Lys B:393, His B:359, Asn B117 (2), Gly B:118, Thr B:120
Isorhamnetin 3‐O‐glucoside	*MurE of E. coli*	1e8c	−9.4	0.68	H‐bond	9	Glu B:182, Thr B:116, His B:359 (2), Gly B:464, Asp B:413, Asn B:414, His B:210, Tyr B:470
Quercetin 3‐O‐glucoside	*30S ribosome S3 of E. coli*	4v53	−8.3	0.15	H‐bond	5	Lys B:15 (2), Asp B:182, Asp B:111 (2)
3‐O‐Caffeoylquinic acid	*30S ribosome S3 of E. coli*	4v53	−8.7	0.00	H‐bond	3	Lys B:107, Asn B:7, Asp B:182
5‐O‐Caffeoylquinic acid	*30S ribosome S3 of E. coli*	4v53	−8.6	0.00	H‐bond	3	Asn B:7, Asp B:182, Lys B:15
Isorhamnetin 3‐O‐rutinoside	*30S ribosome S3 of E. coli*	4v53	−8.4	8.84	H‐bond	4	Asn B:18, Asn B:184 (3)
Rutin	*30S ribosome S3 of E. coli*	4v53	−8.7	0.42	H‐bond	4	Asp B:117, Ala B:47, Glu B:109, Gln B:122
Isorhamnetin 3‐O‐glucoside	*30S ribosome S3 of E. coli*	4v53	−8.1	0.99	H‐bond	2	Tyr B:183, Asn B:7
Quercetin 3‐O‐glucoside	*Transpeptidase of E. coli*	6ntw	−8.4	1.03	H‐bond	3	Gly A:582, Gln A:394, Asp A:397
Isorhamnetin 3‐O‐rutinoside	*Transpeptidase of E. coli*	6ntw	−8.7	1.02	H‐bond	8	Gln A:588, Ser A:398, Arg A:590, Ala A:383, Tyr A:384, Asp A:520 (2), Ser A:385
Rutin	*Transpeptidase of E. coli*	6ntw	−8.6	0.88	H‐bond	6	Ala A:383, Arg A:590 (2), Ala A:583, Arg A:522 (2)
Isorhamnetin	*Gyrase B of E. coli*	1kzn	−8.1	1.06	H‐bond	0	
Quercetin 3‐O‐glucoside	*Gyrase B of E. coli*	1kzn	−8.1	0.88	H‐bond	6	Ile A:90, Val A:120, Asp A:73, Glu A:50, Arg A:76 (2)
5‐O‐Caffeoylquinic acid	*Gyrase B of E. coli*	1kzn	−8.1	0.55	H‐bond	5	Ile A:90 Val A:120, Asp A:73 (2), Asn A:46
Isorhamnetin 3‐O‐rutinoside	*Gyrase B of E. coli*	1kzn	−8.2	0.77	H‐bond	4	Glu A:50 (2), Ser A:121, Val A:118
Rutin	*Gyrase B of E. coli*	1kzn	−8.2	0.58	H‐bond	5	Arg A:136, Gly A:77, Asn A:46, Arg A:76, Asp A:73
Isorhamnetin 3‐O‐glucoside	*Gyrase B of E. coli*	1kzn	−8.0	0.91	H‐bond	1	Arg A:76
Quercetin 3‐O‐glucoside	*Dihydropteroate synthase of E. coli*	5v7a	−8.8	0.30	H‐bond	2	Arg A:63, Asp A:96
3‐O‐Caffeoylquinic acid	*Dihydropteroate synthase of E. coli*	5v7a	−8.1	4.57	H‐bond	5	Phe A:188, Thr A:62, Asn A:115, Asp A:185, Arg A:63
5‐O‐Caffeoylquinic acid	*Dihydropteroate synthase of E. coli*	5v7a	−8.2	1.05	H‐bond	2	Asn A:115, Thr A:62
Isorhamnetin 3‐O‐rutinoside	*Dihydropteroate synthase of E. coli*	5v7a	−8.0	6.17	H‐bond	4	Arg A:235 (2), Glu A:60, Asp A:96
Rutin	*Dihydropteroate synthase of E. coli*	5v7a	−8.1	0.84	H‐bond	4	His A:257, Arg A:235, Thr A:62 (2)
Isorhamnetin 3‐O‐glucoside	*Dihydropteroate synthase of E. coli*	5v7a	−8.9	0.00	H‐bond	2	Gly A:189, Arg A:255

**FIGURE 3:**
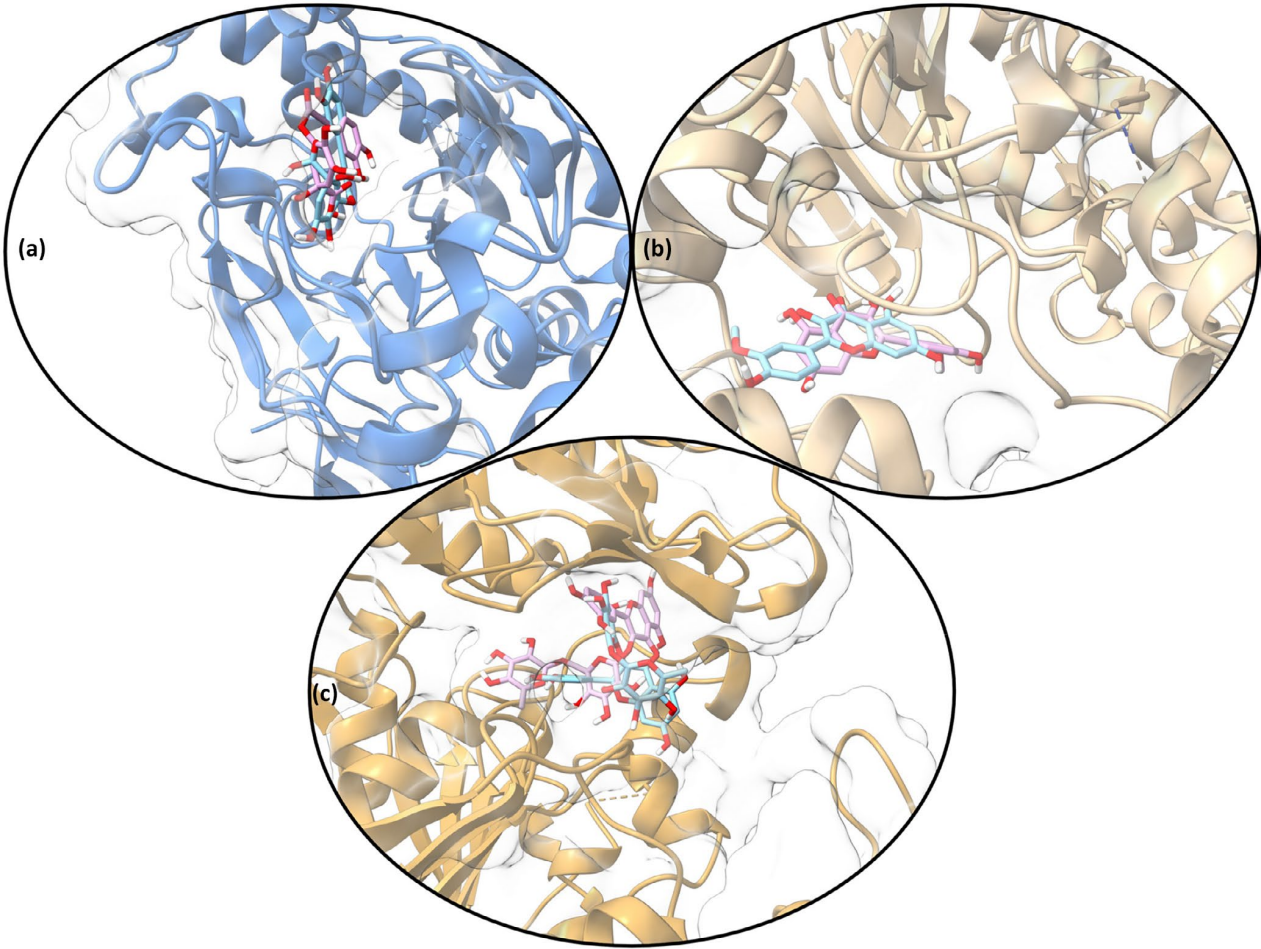
Molecular interactions with high RMSD values.

Interactions with binding energies of −8 kcal/mol or lower were found to have RMSD values ranging from 0 to 8.53 Å. An RMSD value greater than 2 Å is indicative of unreliable results, as such values may be indicative of a lack of precision in the data (Table 5). For example, the RMSD was found to be 5.32 Å in the interaction between 5‐O‐caffeoylquinic acid and BChE (Figure 3a). Similarly, the interaction between isorhamnetin and the MurE enzyme of *S. aureus* resulted in an RMSD of 8.53 Å (Figure 3b). Despite the interaction between rutin and the MurE enzyme of *S. aureus* exhibiting 11 hydrogen bonds with a binding energy of −10.9 kcal/mol, the RMSD was determined to be 6.73 Å (Figure 3c). The RMSD values suggest that the interactions are not stable, which raises concerns about the reliability of the observed binding modes.

The antimicrobial effects of hub molecules derived from *R. turkestanicum* and *C. officinalis* on various bacterial enzymes, proteins, and standard enzymes were comprehensively evaluated, with 11 complexes identified as particularly significant. The selection of these complexes was based on key parameters such as binding energies, hydrogen bond counts, and RMSD values. For example, the rutin molecule exhibited binding energy of −9.4 kcal/mol with amylase and an RMSD value of 0.8589 Å, forming six hydrogen bonds with residues His A:305, Asp A:197, Glu A:233, and Lys A:200. Quercetin 3‐O‐glucoside showed binding energy of −11.0 kcal/mol with AChE and an RMSD value of 1.0523 Å, interacting with residues Tyr A:133, Glu A:202, Ala A:204, and Tyr A:341 through four hydrogen bonds. Similarly, quercetin 3‐O‐glucoside demonstrated binding energy of −10.1 kcal/mol with BChE (3djy) and an RMSD value of 0.9764 Å, forming eight hydrogen bonds with residues Gly A:116 (twice), Trp A:82, Gly A:115 (twice), Tyr A:128, Asp A:70, and Tyr A:332. The rutin molecule also displayed a binding energy of −9.0 kcal/mol with Tyr (5m8o) and an RMSD value of 0.1981 Å, interacting with residues Thr A:391, Asp A:212, Thr A:362, Asn A:378, and Ser A:394 through five hydrogen bonds. Furthermore, 3‐O‐Caffeoylquinic acid exhibited a binding energy of −9.8 kcal/mol with MurE of *S. aureus* (4c13) and an RMSD value of 0.0392 Å, forming 11 hydrogen bonds with residues Thr A:115, Lys A:114 (twice), His A:205, Thr A:111, Asn A:301, Ser A:116, His A:353 (twice), and Gly A:113 (twice). Isorhamnetin 3‐O‐glucoside demonstrated a binding energy of −9.1 kcal/mol with the same enzyme (4c13), with an RMSD value of 0.1382 Å, forming eight hydrogen bonds with residues Ser A:456, Asp A:406, Arg A:383 (twice), Ala A:150, Asn A:151, and Thr A:115. Additionally, isorhamnetin 3‐O‐glucoside showed a binding energy of −8.7 kcal/mol with transpeptidase of *S. aureus* (5tw8) and an RMSD value of 0.9367 Å, forming eight hydrogen bonds with residues Glu A:297, Thr A:260, Ser A:75, Ser A:139 (twice), Ser A:116, Asn A:141, and Glu A:114. The rutin molecule displayed binding energy of −10.6 kcal/mol with MurE of *E. coli* (1e8c) and an RMSD value of 1.0839 Å, forming 11 hydrogen bonds with residues Lys B:119 (three times), Glu B:155, Arg B:389, Lys B:393, His B:359, Asn B:117 (twice), Gly B:118, and Thr B:120. Isorhamnetin 3‐O‐glucoside, with the same enzyme (1e8c), exhibited a binding energy of −9.4 kcal/mol and an RMSD value of 0.683 Å, forming nine hydrogen bonds with residues Glu B:182, Thr B:116, His B:359 (twice), Gly B:464, Asp B:413, Asn B:414, His B:210, and Tyr B:470. Quercetin 3‐O‐glucoside demonstrated a binding energy of −8.3 kcal/mol with *E. coli* 30S ribosome S3 (4v53) and an RMSD value of 0.1455 Å, forming five hydrogen bonds with residues Lys B:15 (twice), Asp B:182, and Asp B:111 (twice). Finally, rutin, with the same enzyme (4v53), exhibited a binding energy of −8.7 kcal/mol and an RMSD value of 0.4176 Å, forming four hydrogen bonds with residues Asp B:117, Ala B:47, Glu B:109, and Gln B:122. These findings, consistent with previous literature, demonstrate the high binding stability and strong inhibitory potential of the selected molecules. The molecular docking analyses revealed that bioactive compounds such as isorhamnetin, quercetin 3‐O‐glucoside, 3‐O‐caffeoylquinic acid, 5‐O‐caffeoylquinic acid, isorhamnetin 3‐O‐rutinoside, and rutin, derived from these plants, possess high antimicrobial and standard enzymes potential. These compounds may be effective through the inhibition of bacterial enzymes proteins, and standard enzymes making them prominent in our analyses.

### 3.9 MM/PBSA Binding Free Energy Calculation

In this study, the antimicrobial activities of phenolic and flavonoid compounds derived from R. *turkestanicum* and *C. officinalis* were analyzed using MD simulations and MM/PBSA binding free energy calculations. Based on criteria such as low RMSD, high binding energy, and the number of hydrogen bonds, 11 complexes were selected for further analysis. These selected complexes include: *E. coli* MurE_isorhamnetin 3‐O‐glucoside, *E. coli* MurE_rutin, Amylase_rutin, AChE_quercetin 3‐O‐glucoside, BChE_isorhamnetin 3‐O‐rutinoside, BChE_quercetin 3‐O‐glucoside, *S. aureus* MurE_3‐O‐caffeoylquinic acid, *S. aureus* MurE_isorhamnetin 3‐O‐glucoside, *E. coli* 30S ribosome S3_quercetin 3‐O‐glucoside, Tyr_rutin, and *E. coli* Transpeptidase_isorhamnetin 3‐O‐glucoside (Table S3).

**FIGURE 4:**
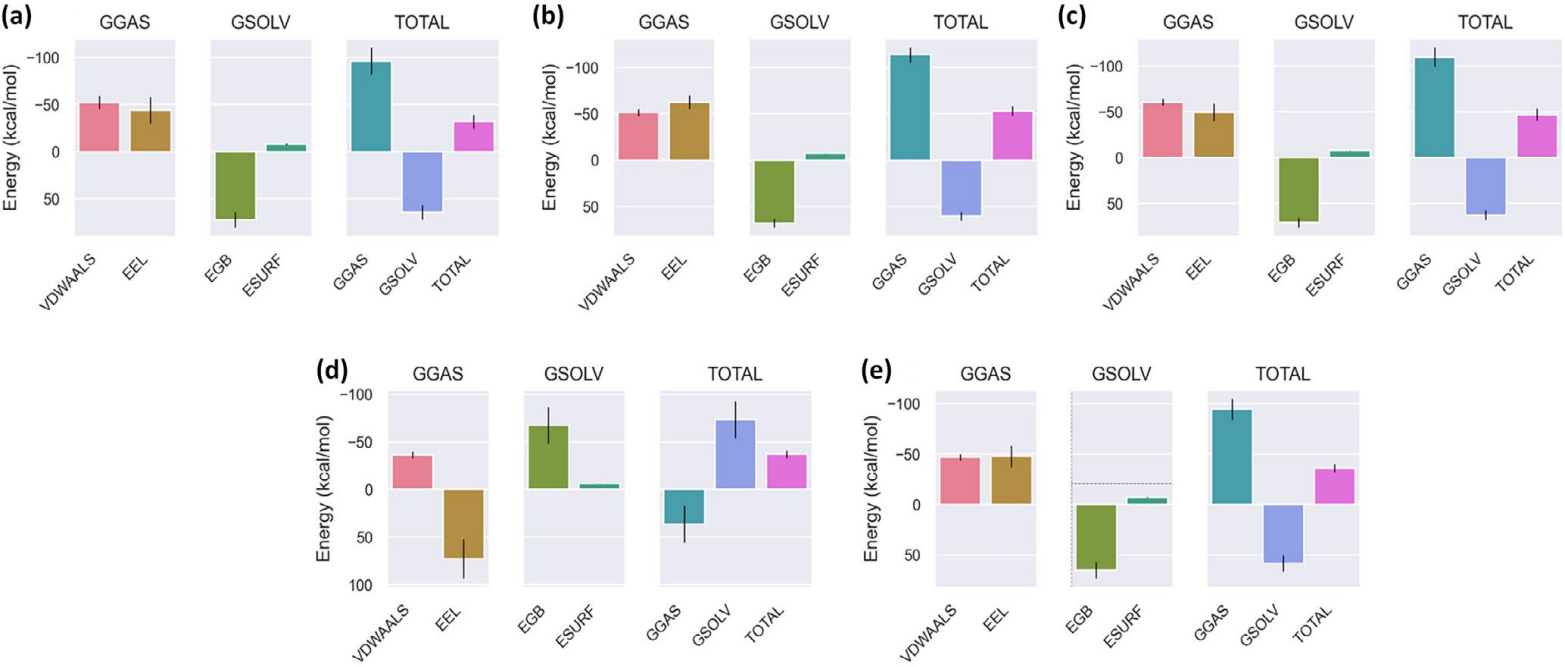
MM/PBSA binding free energy analysis (a) *E. coli* MurE_rutin complex. (b) AChE_quercetin 3‐O‐glucoside complex. (c) BChE_isorhamnetin 3‐O‐rutinoside complex. (d) *S. aureus* MurE_3‐O‐caffeoylquinic acid complex. (e) *S. aureus* MurE_isorhamnetin 3‐O‐glucoside complex.

Examining the energy components, for the *E. coli* MurE_isorhamnetin 3‐O‐glucoside complex, the van der Waals interaction energy (VDWAALS) was calculated to be −30.92 kcal/mol (SD = 6.88), the electrostatic energy (EEL) as −67.29 kcal/mol (SD = 24.13), the polar solvation energy (EGB) as 79.62 kcal/mol (SD = 15.64), and the surface tension energy (ESURF) as −5.79 kcal/mol (SD = 0.96). The total energy was determined to be −24.38 kcal/mol (SD = 13.07). For the *E. coli* MurE_rutin complex, the van der Waals energy was −52.2 kcal/mol (SD = 6.98), the electrostatic energy was −44.15 kcal/mol (SD = 14.09), the polar solvation energy was 72.26 kcal/mol (SD = 8.23), and the surface tension energy was −7.88 kcal/mol (SD = 0.78), with a total energy of −31.96 kcal/mol (SD = 7.37). Similarly, in the amylase_rutin complex, the van der Waals energy was −48.71 kcal/mol (SD = 3.24), the electrostatic energy was −28.61 kcal/mol (SD = 3.95), the polar solvation energy was 53.67 kcal/mol (SD = 3.17), and the surface tension energy was −6.75 kcal/mol (SD = 0.36), for total energy of −30.39 kcal/mol (SD = 3.83). The total energy for the AChE_quercetin 3‐O‐glucoside complex was −53.08 kcal/mol (SD = 5.19), while for the BChE_isorhamnetin O‐rutinoside complex it was calculated to be −46.83 kcal/mol (SD = 6.69) (Figure 4) (Table S3). These energy components indicate that the compounds exhibit strong binding stability and high affinity for their enzyme/protein targets. The data indicate that rutin, isorhamnetin, and quercetin derivatives should be considered as potential inhibitors and serve as a valuable basis for drug discovery studies.

### 3.10 MD Simulation

**FIGURE 5:**
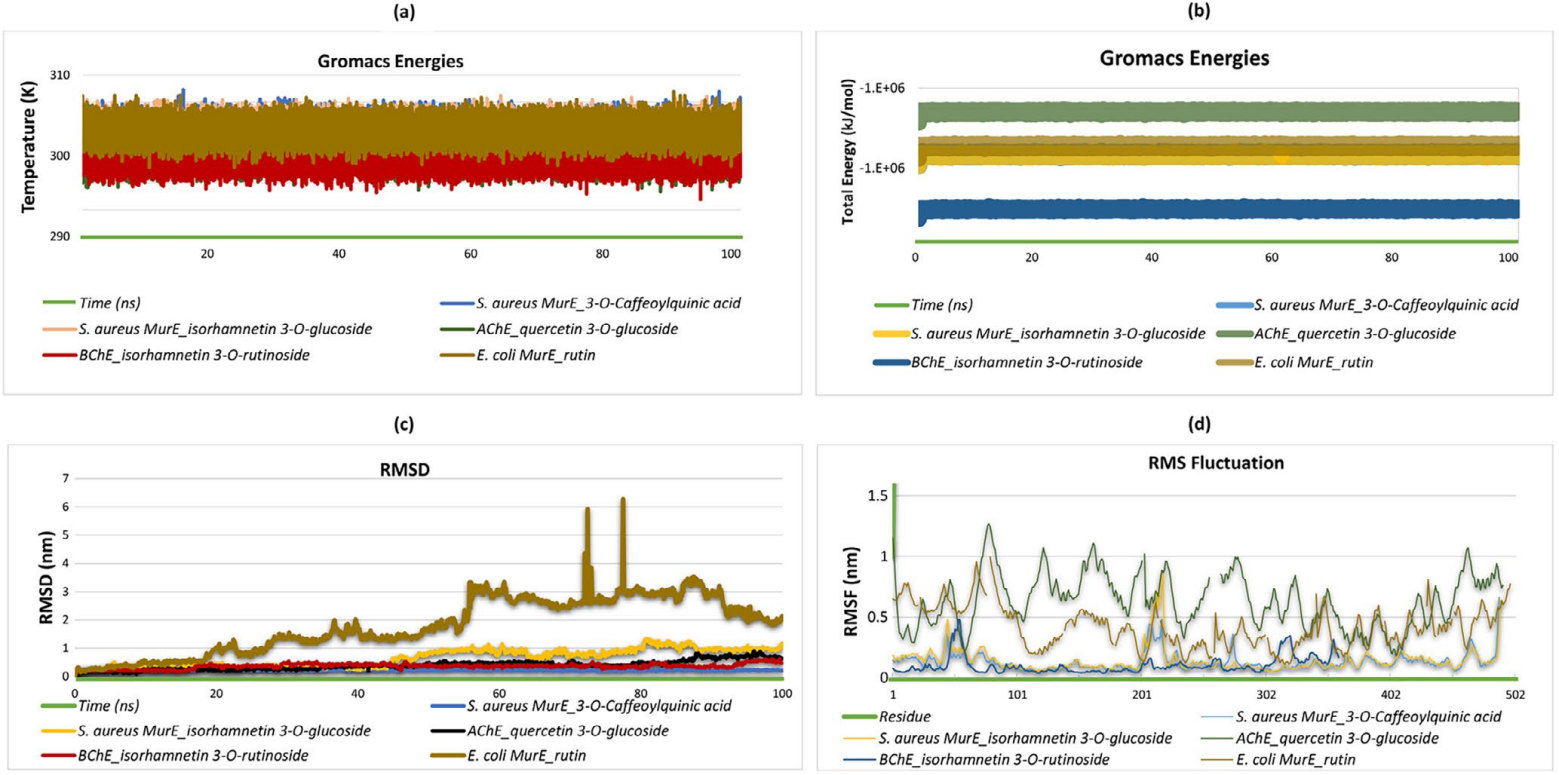
Presentation of molecular dynamics simulations in graphical form. (a) Total energy of *S. aureus* MurE—isorhamnetin 3‐O‐glucoside, *S. aureus* MurE—3‐O‐caffeoylquinic acid, AChE—quercetin 3‐O‐glucoside, BChE—isorhamnetin 3‐O‐rutinoside, and *E. coli* MurE—rutin complexes. (b) Temperature of *S. aureus* MurE—isorhamnetin 3‐O‐glucoside, *S. aureus* MurE—3‐O‐caffeoylquinic acid, AChE—quercetin 3‐O‐glucoside, BChE—isorhamnetin 3‐O‐rutinoside, and *E. coli* MurE—rutin complexes. (c) RMSD of *S. aureus* MurE—isorhamnetin 3‐O‐glucoside, *S. aureus* MurE—3‐O‐caffeoylquinic acid, AChE—quercetin 3‐O‐glucoside, BChE—isorhamnetin 3‐O‐rutinoside, and *E. coli* MurE—rutin complexes. (d) *S. aureus* MurE—isorhamnetin 3‐O‐glucoside, *S. aureus* MurE—3‐O‐caffeoylquinic acid, AChE—quercetin 3‐O‐glucoside, BChE—isorhamnetin 3‐O‐rutinoside, and *E. coli* MurE—rutin complexes.

The aim of this study is to identify potential therapeutic agents by thoroughly investigating the molecular interactions between ligands and their target proteins. A detailed analysis of nine potential ligands was performed to evaluate their biological efficacy and protein binding capabilities. The ligands were initially selected based on their molecular docking scores, followed by further refinement using MM/PBSA binding free energy calculations. From this analysis, the top five complexes were selected for MD simulations: rutin with *E. coli* MurE, quercetin 3‐O‐glucoside with AChE, isorhamnetin 3‐O‐rutinoside with BChE, caffeoylquinic acid with *S. aureus* MurE, and isorhamnetin 3‐O‐glucoside with *S. aureus* MurE. These ligands exhibited strong interaction metrics in vitro and were selected based on their binding specificity and the stability of their interactions with the target proteins as revealed by energy calculations. The temperature and energy profiles for all complexes remained stable around 300 K, indicating that the system reached thermal equilibrium and that the protein–ligand interactions were structurally stable (Figure 5a,b). These results suggest that the simulations are biologically reliable and reproducible.

The RMSD plot illustrates how the RMSD values change over time for each ligand. The RMSD values for 3‐O‐caffeoylquinic acid and isorhamnetin 3‐O‐glucoside with *S. aureus* MurE, as well as quercetin 3‐O‐glucoside with AChE and Isorhamnetin 3‐O‐rutinoside with BChE, follow a relatively lower and more stable trend. In contrast, Rutin with *E. coli* MurE exhibits a higher and more fluctuating RMSD profile, where the values rise to between 0 and 3 nm up to 50 ns, but after 90 ns, the RMSD decreases to around 2 nm and stabilizes. (Figure 5c). This study examines the RMSF analysis of five different protein‐ligand complexes and correlates these data with docking results. In the isorhamnetin 3‐O‐glucoside‐MurE of *S. aureus* complex, a significant increase in RMSF values is observed in the residue range of 100–200, indicating greater flexibility in this region of the protein. In these flexible regions, the ligand interacts with residues Ser A:456 (0.0999 nm), Asp A:406 (0.1547 nm), Arg A:383 (0.2003 nm), Ala A:150 (0.1462 nm), Asn A:151 (0.114 nm), and Thr A:115 (0.0678 nm). For the 3‐O‐caffeoylquinic acid‐MurE of *S. aureus* complex, a noticeable increase in RMSF values is observed between residues 300 and 500, corresponding to interactions between the ligand and residues Thr A:115 (0.0631 nm), Lys A:114 (0.0629 nm), His A:205 (0.2628 nm), Thr A:111 (0.0711 nm), Asn A:301 (0.0806 nm), Ser A:116 (0.062 nm), His A:353 (0.0703 nm), and Gly A:113 (0.0695 nm). The quercetin 3‐O‐glucoside‐AChE complex shows the highest RMSF values in the residue ranges of 100–200 and 400–500, where the ligand interacts with residues Tyr A:133 (0.7663 nm), Glu A:202 (0.9641 nm), Ala A:204 (0.5853 nm), and Tyr A:341 (0.6383 nm). The isorhamnetin 3‐O‐rutinoside‐BChE complex exhibits generally low RMSF values, indicating greater protein stability, with the ligand binding to residues Leu A:76 (0.3886 nm), Ser A:293 (0.057 nm), and Tyr A:341 (0.3298 nm). Finally, in the Rutin‐MurE of *E. coli* complex, RMSF values increase significantly after residue 300, where the ligand interacts with residues Lys B:119 (0.1918 nm), Glu B:155 (0.5627 nm), Arg B:389 (0.4633 nm), Lys B:393 (0.5963 nm), His B:359 (0.2079 nm), Asn B:117 (0.2108 nm), Gly B:118 (0.1919 nm), and Thr B:120 (0.205 nm). These results suggest that the flexible regions of the protein are closely associated with ligand binding sites and that RMSF values play a critical role in understanding the structural dynamics of protein–ligand interactions (Figure 5c).

**FIGURE 6:**
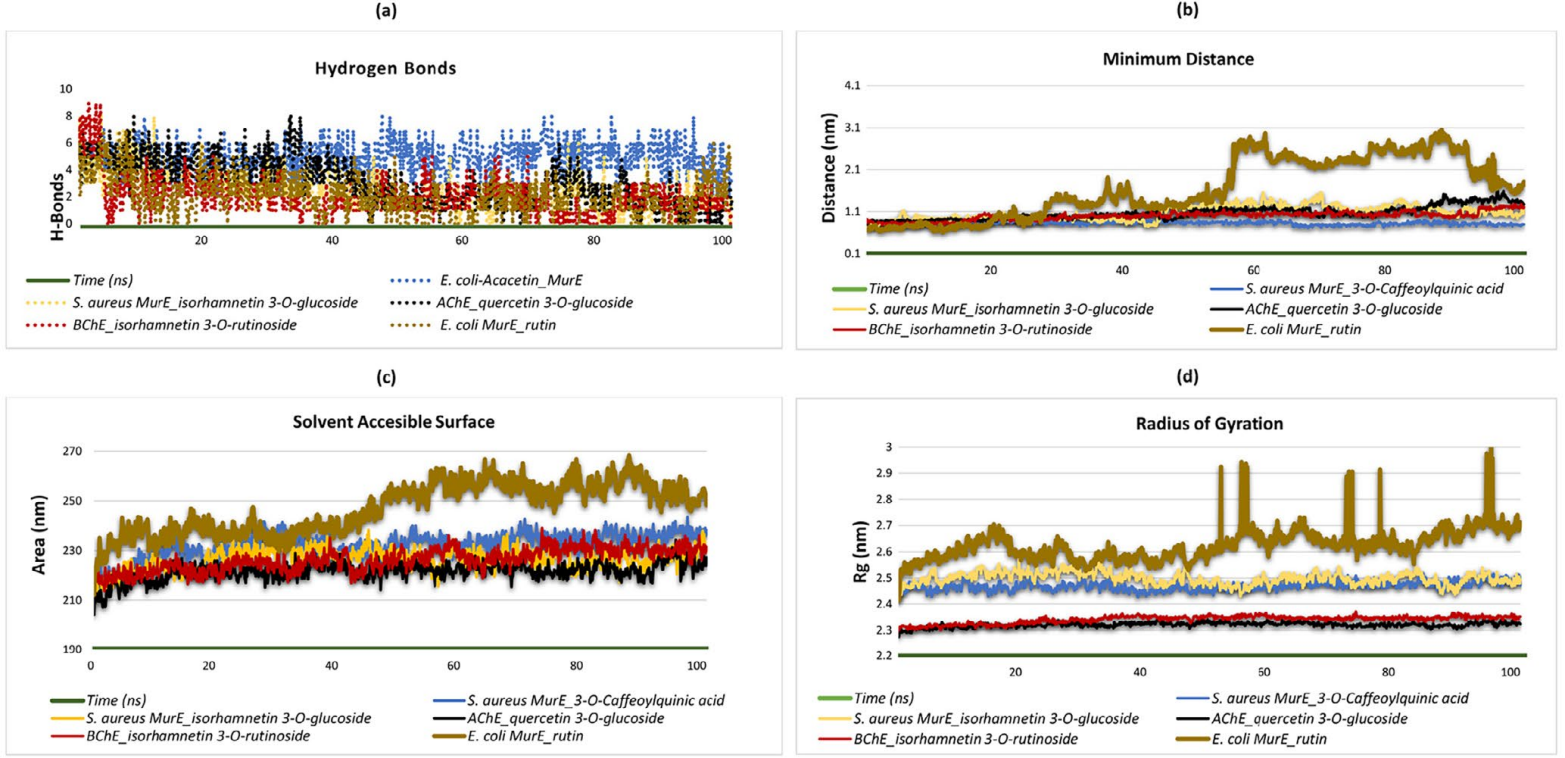
Presentation of molecular dynamics simulations in graphical form. (a) Minimum distance of *S. aureus* MurE—isorhamnetin 3‐O‐glucoside, *S. aureus* MurE—3‐O‐caffeoylquinic acid, AChE—quercetin 3‐O‐glucoside, BChE—isorhamnetin 3‐O‐rutinoside, and *E. coli* MurE—rutin complexes. (b) Hydrogen bonds in *S. aureus* MurE—isorhamnetin 3‐O‐glucoside, *S. aureus* MurE—3‐O‐caffeoylquinic acid, AChE—quercetin 3‐O‐glucoside, BChE—isorhamnetin 3‐O‐rutinoside, and *E. coli* MurE—rutin complexes. (c) Solvent accessibility of *S. aureus* MurE—isorhamnetin 3‐O‐glucoside, *S. aureus* MurE—3‐O‐caffeoylquinic acid, AChE—quercetin 3‐O‐glucoside, BChE—isorhamnetin 3‐O‐rutinoside, and *E. coli* MurE—rutin complexes. (d) Radius of gyration of *S. aureus* MurE—isorhamnetin 3‐O‐glucoside, *S. aureus* MurE—3‐O‐caffeoylquinic acid, AChE—quercetin 3‐O‐glucoside, BChE—isorhamnetin 3‐O‐rutinoside, and *E. coli* MurE—rutin complexes.

MD simulations are a vital instrument for elucidating the dynamics of hydrogen bonding and the minimal distances in protein–ligand complexes. The findings from these simulations indicate that the isorhamnetin 3‐O‐glucoside‐ *S. aureus* MurE complex forms between two and eight hydrogen bonds, with an average of three. However, there are occasional fluctuations in this number. As the simulation progresses, the number of hydrogen bonds stabilizes between zero and two. The 3‐O‐caffeoylquinic acid‐MurE complex of *S. aureus* reaches a maximum of eight hydrogen bonds and generally remains stable around four bonds. By the conclusion of the simulation, the complex exhibits a stabilization of four to six hydrogen bonds. The quercetin 3‐O‐glucoside‐AChE complex forms between zero and eight hydrogen bonds, with an average of three. However, fluctuations are observed. As the simulation progresses, the number of hydrogen bonds decreases and ultimately stabilizes between zero and two. The isorhamnetin 3‐O‐rutinoside‐BChE complex exhibits a hydrogen bond range between zero and nine, with an average of two bonds. By the conclusion of the simulation, the bond count stabilizes between zero and two. The rutin‐MurE of the *E. coli* complex forms between zero and seven hydrogen bonds, with an average of two. Toward the conclusion of the simulation, the number of hydrogen bonds stabilizes between zero and four. These results underscore the dynamic nature of hydrogen bond interactions in these protein–ligand complexes, offering valuable insights into their stability and binding behavior throughout the MD simulations (Figure 6a).

The data obtained from MD simulations offer substantial insights into the minimum distances of protein–ligand complexes. In the isorhamnetin 3‐O‐glucoside‐ *S. aureus* MurE complex, the minimum distances ranged from 0.71 to 1.5 nm, with an average value of 1.07 nm, and remained stable throughout the simulation. Similarly, the 3‐O‐caffeoylquinic acid‐MurE complex *of S. aureus* complex exhibited minimum distances between 0.79 and 1.5 nm, with an average of 0.82 nm, demonstrating consistent stability. The quercetin 3‐O‐glucoside‐AChE complex exhibited minimum distances ranging from 0.79 to 1.5 nm, with an average of 1.06 nm, and demonstrated consistent stability. In the isorhamnetin 3‐O‐rutinoside‐BChE complex, the minimum distances exhibited a range of 0.78–1.2 nm, with an average of 0.9 nm. However, throughout the simulation, these distances remained stable. In contrast, the Rutin‐MurE of the *E. coli* complex exhibited notable variation in minimum distances, ranging from 0.62 to 3.01 nm, with an average of 1.6 nm. This indicates fluctuating interactions compared to the more stable distances observed in the other complexes. The results of this analysis demonstrate that, with the exception of the Rutin‐MurE of the *E. coli* complex, all complexes exhibited relatively stable minimum distances throughout the course of the simulations (Figure 6b).

MD simulations are an invaluable tool for elucidating the structural dynamics of protein–ligand complexes and their interactions with the solvent. In this study, the solvent‐accessible surface area (SASA) and radius of gyration (Rg) were analyzed to assess the stability and conformational changes of the complexes. Figure 6c,d illustrate the variations in these properties. The data demonstrate that the isorhamnetin 3‐O‐glucoside‐ *S. aureus* MurE complex exhibited SASA values that ranged from 207.539 to 238.409 nm^2^, with an average of 226.391 nm^2^. The complex initially exhibited elevated Rg values, which subsequently transitioned toward a more stable conformation over time, resulting in an average Rg value of 2.50 nm. Similarly, the 3‐O‐caffeoylquinic acid‐MurE complex of *S. aureus* demonstrated SASA values between 206.913 and 243.489 nm^2^, with an average of 233.857 nm^2^. Additionally, the complex initially exhibited elevated Rg values, which stabilized over time, resulting in an average Rg value of 2.47 nm. The quercetin 3‐O‐glucoside‐AChE complex exhibited SASA values that ranged from 204.261 to 255.623 nm^2^, with an average of 230.462 nm^2^. The Rg values exhibited a comparable pattern, demonstrating stabilization over time with an average Rg value of 2.32 nm. The isorhamnetin 3‐O‐rutinoside‐BChE complex exhibited SASA values between 155.823 and 216.56 nm^2^, with an average of 226.366 nm^2^. Its Rg values stabilized at an average of 2.63 nm after an initial period of fluctuation. Lastly, the complex of rutin and MurE from *E. coli* exhibited SASA values ranging from 212.223 to 260.646 nm^2^, with an average of 247.133 nm^2^. Similarly, this complex initially exhibited elevated Rg values, which subsequently transitioned to a more stable conformation, with an average Rg value of 2.47 nm. These findings suggest that while all complexes initially demonstrated fluctuations in Rg values, they subsequently exhibited a transition toward more stable conformations over time, as evidenced by their respective SASA and Rg values.

The complexes of isorhamnetin 3‐O‐glucoside‐ *S. aureus* MurE, 3‐O‐caffeoylquinic acid‐MurE of *S. aureus* , and quercetin 3‐O‐glucoside‐AChE were identified as the most promising drug candidates based on the results of the minimum distance, hydrogen bond stability, RMSD, RMSF, Rg, and SASA analyses. The isorhamnetin 3‐O‐rutinoside‐BChE complex also exhibits notable stability, whereas the rutin‐MurE of the *E. coli* complex displays a comparatively less stable profile.

## 4 Conclusions

The study's results provide insights into the unique profiles of phenolic compounds and flavonoids present in infusion and ethanolic extracts of *R. turkestanicum* and *C. officinalis* . Notably, a higher concentration of phenolics was observed in the ethanolic extract of *R. turkestanicum* with a value of 75.82 ± 0.18 mg GAE/g, while flavonoids were more abundant in the infusion extract of *C. officinalis* . A total of 20 bioactive compounds were identified in the tested extracts. The ethanol extracts of both Calendula flowers and R. tanguticum demonstrated significantly higher antioxidant activities compared to their respective infusions, The ethanol extracts of COF and RT exhibited superior enzyme inhibitory activities compared to their respective infusions, with notable increases in AChE, BChE, and tyrosinase inhibition. Ethanol extracts generally exhibited lower MIC and MBC values compared to their respective infusions. In silico molecular docking and MD simulations provide further evidence to support the bioactivity potential of these extracts. In silico studies demonstrated that multiple bioactive compounds derived from both COF and RT extracts exhibited stable interactions with pivotal target proteins, including AChE, BChE, and MurE. These proteins play crucial roles in neurodegenerative diseases and bacterial cell wall synthesis. MD simulations confirmed the stability of these interactions, with particularly promising results observed for isorhamnetin 3‐O‐glucoside and 3‐O‐caffeoylquinic acid in their respective target proteins and enzymes. These compounds demonstrated strong binding affinities and stable hydrogen bond formations, suggesting their potential as therapeutic agents. Hence, there is a need to isolate and identify compounds in various active extracts for additional investigation into their activities and mode of operation. These plant species could potentially serve as a valuable reservoir of bioactive compounds for the food industry, especially in the creation of innovative functional foods and/or dietary supplements.

## Author Contributions

**Serdar Korpayev:** conceptualization (equal), data curation (equal), resources (equal), supervision (equal). **Gokhan Zengin:** conceptualization (equal), data curation (equal), investigation (equal), methodology (equal), writing – original draft (equal), writing – review and editing (equal). **Gunes Ak:** conceptualization (equal), investigation (equal), methodology (equal), supervision (equal), writing – original draft (equal), writing – review and editing (equal). **Jasmina Glamočlija:** conceptualization (equal), investigation (equal), methodology (equal), writing – original draft (equal), writing – review and editing (equal). **Marina Soković:** investigation (equal), methodology (equal), writing – original draft (equal), writing – review and editing (equal). **Neda Aničić:** investigation (equal), methodology (equal), writing – original draft (equal), writing – review and editing (equal). **Uroš Gašić:** conceptualization (equal), investigation (equal), methodology (equal), writing – original draft (equal), writing – review and editing (equal). **Dejan Stojković:** formal analysis (equal), investigation (equal), methodology (equal), writing – original draft (equal), writing – review and editing (equal). **Mirap Agamyradov:** investigation (equal), methodology (equal), writing – original draft (equal), writing – review and editing (equal). **Mehmet Veysi Cetiz:** conceptualization (equal), data curation (equal), visualization (equal), writing – original draft (equal). **Guljan Agamyradova:** investigation (equal), methodology (equal), writing – original draft (equal), writing – review and editing (equal).

## Ethics Statement

The authors have nothing to report.

## Consent

Written informed consent was obtained from all study participants.

## Conflicts of Interest

The authors declare no conflicts of interest.

## Supporting information


Data S1.

## Data Availability

The data that support the findings of this study are available on request from the corresponding author.
