# Supplementary material for: Integration of In Vitro and In Silico Results From Chemical and Biological Assays of Rheum turkestanicum and Calendula officinalis Flower Extracts
Source: Food Sci Nutr. 2024 Dec 19;13(1):e4663. doi: 10.1002/fsn3.4663 (PMC11717056; doi:10.1002/fsn3.4663)
Supplement: Supplementary file 1 — Data S1. [file FSN3-13-e4663-s001.docx]

**Integration of *in vitro* and *in silico* results from chemical and biological assays of *Rheum turkestanicum* and *Calendula officinalis* flower extracts**

Serdar Korpayev^1^, Gokhan Zengin^2*^, Gunes Ak^2^, Jasmina Glamočlija^3^, Marina Soković^3^, Neda Aničić^3^, Uroš Gašić^3^, Dejan Stojković^3^, Mirap Agamyradov^4^, Mehmet Veysi Cetiz^2,5,6^, Guljan Agamyradova^4^

*^1^Biotechnology Institute, Ankara University, 06100 Ankara, Turkey*

*^2^Department of Biology, Science Faculty, Selcuk University, Konya, Turkey*

*^3^Department of Plant Physiology, Institute for Biological Research “Siniša Stanković” - National Institute of Republic of Serbia, University of Belgrade, Bulevar Despota Stefana 142, 11108 Belgrade, Serbia*

*^4^Saint Petersburg State Pediatric Medical University,*

*^5^Cetiz Lab. Sanlıurfa, 63600, Turkiye*

*^6^Department of Chemistry, Recep Tayyip Erdogan University, 53100, Rize, Turkey*

**Correspondence:** gokhanzengin@selcuk.edu.tr

**Supplementary Tables**

**Table S1.** Relevant protein and enzyme target coordinates of the docking box.

| **Receptor** | | | | **Coordinate** | | **Reference** |
| --- | --- | --- | --- | --- | --- | --- |
| **Target** | | | **PDB ID** | **Grid size X, Y, Z** | **X, Y, Z dimensions** |  |
| **Enzymes** | AChE | 2y2v | | 22 Å X 30 Å X 40 Å | 31.062, 20.311, 11.947 | (Yagi et al., 2024} |
|  | BChE | 3djy | | 30 Å X 30 Å X 30 Å | 44.794, -19.63, -25.227 | (Duran et al., 2024) |
|  | Tyr | 5m8o | | 26 Å X 26 Å X 28 Å | -13.194, 5.341, -26.28 | (Yagi et al., 2024 |
|  | Amylase | 2qv4 | | 28 Å 28 Å X 24 Å | 14.188, 48.964, 22.886 | (Yagi et al., 2024) |
|  | Glucosidase | 3w37 | | 42 Å 52 Å X 54 Å | 3.091, −8.008, −4.08 | (Duran et al., 2024) |
| ***S. aureus*** | 30S ribosome S3 | 5tcu | | 76 Å x 76 Å x 76 Å | 99.46, 230.082, 201.387 | (Saqallah et al., 2022) |
|  | Dihydropteroate synthase | 1ad4 | | 60 Å x 60 Å x 60 Å | 32.46, 6.683, 42,972 | (Saqallah et al., 2022; Yu et al., 2010) |
|  | Gyrase B | 4urn | | 60 Å x 60 Å x 40 Å | -31.684, -5.252, 1.572 | (Saqallah et al., 2022; Yu et al., 2010) |
|  | MurE | 4c13 | | 60 Å x 60 Å x 60 Å | -23.122, 2.508, 9.873 | (Saqallah et al., 2022; Yu et al., 2010) |
|  | Transpeptidase | 5tw8 | | 60 Å x 60 Å x 60 Å | 21.390, -62.210, 39.196 | (Saqallah et al., 2022) |
| ***E. coli*** | 30S ribosome S3 | 4v53 | | 88 Å x 88 Å x 88 Å | 130.966, 32.099, 0.385 | (Saqallah et al., 2022; Yu et al., 2010) |
|  | Dihydropteroate synthase | 5v7a | | 60 Å x 60 Å x 60 Å | -17.836, -17.836, 103.740 | (Saqallah et al., 2022) |
|  | Gyrase B | 1kzn | | 60 Å x 60 Å x 60 Å | 12.467, 27.336, 44.916 | (Saqallah et al., 2022; Yu et al., 2010) |
|  | MurE | 1e8c | | 70 Å x 60 Å x 70 Å | 45.098, 37.112, 76.674 | (Saqallah et al., 2022; Yu et al., 2010) |
|  | Transpeptidase | 6ntw | | 60 Å x 60 Å x 60 Å | 16.929, -32.370, 42.151 | (Saqallah et al., 2022; Yu et al., 2010) |

**Table S2** Relevant protein and enzyme result of the docking scores

| **Compound and Receptor**  **Receptor** | | | | **Binding energy** | | **Receptor** | | **Binding energy** | **Receptor** | | **Binding energy** | | **Receptor** | **Binding energy** | | **Receptor** | **Binding energy** |
| --- | --- | --- | --- | --- | --- | --- | --- | --- | --- | --- | --- | --- | --- | --- | --- | --- | --- |
| **Compound** | | **PDB ID** | |  |  | **PDB ID** | |  | **PDB ID** | |  |  | **PDB ID** |  |  | **PDB ID** |  |
| **Enzyme** | isorhamnetin | | 2qv4 | -9.0 | 3w37 | | -7.8 | | 2y2v | -8.8 | | 3djy | | -9.0 | 5m8o | | -6.9 |
|  | p-coumaric acid | | 2qv4 | -6.2 | 3w37 | | -7.8 | | 2y2v | -6.8 | | 3djy | | -6.2 | 5m8o | | -5.8 |
|  | quercetin 3-O-glucoside | | 2qv4 | -8.9 | 3w37 | | -7.8 | | 2y2v | -11.0 | | 3djy | | -10.1 | 5m8o | | -7.6 |
|  | 3-O-Caffeoylquinic acid | | 2qv4 | -8.2 | 3w37 | | -7.8 | | 2y2v | -9.3 | | 3djy | | -8.3 | 5m8o | | -7.7 |
|  | 5-O-Caffeoylquinic acid | | 2qv4 | -8.3 | 3w37 | | -7.8 | | 2y2v | -9.4 | | 3djy | | -8.3 | 5m8o | | -7.8 |
|  | isorhamnetin 3-O-rutinoside | | 2qv4 | -9.6 | 3w37 | | -7.8 | | 2y2v | -10.5 | | 3djy | | -11.0 | 5m8o | | -7.9 |
|  | rutin | | 2qv4 | -9.4 | 3w37 | | -7.8 | | 2y2v | -10.1 | | 3djy | | -10.8 | 5m8o | | -9.0 |
|  | isorhamnetin 3-O-glucoside | | 2qv4 | -8.4 | 3w37 | | -7.8 | | 2y2v | -9.9 | | 3djy | | -10.1 | 5m8o | | -7.3 |
| ***S. aureus*** | isorhamnetin | | 1ad4 | -7.1 | 4urn | | -7.9 | | 5tcu | -6.7 | | 4C13 | | -9.3 | 5TW8 | | -7.9 |
|  | p-coumaric acid | | 1ad4 | -5.5 | 4urn | | -6.0 | | 5tcu | -6.2 | | 4c13 | | -7.7 | 5tw8 | | -5.7 |
|  | quercetin 3-O-glucoside | | 1ad4 | -6.9 | 4urn | | -7.9 | | 5tcu | -7.3 | | 4c13 | | -9.6 | 5tw8 | | -8.9 |
|  | 3-O-Caffeoylquinic acid | | 1ad4 | -7.1 | 4urn | | -7.3 | | 5tcu | -7.4 | | 4c13 | | -9.8 | 5tw8 | | -8.4 |
|  | 5-O-Caffeoylquinic acid | | 1ad4 | -7.9 | 4urn | | -7.5 | | 5tcu | -7.5 | | 4c13 | | -9.2 | 5tw8 | | -8.2 |
|  | isorhamnetin 3-O-rutinoside | | 1ad4 | -8.2 | 4urn | | -7.7 | | 5tcu | -7.8 | | 4c13 | | -10.5 | 5tw8 | | -9.1 |
|  | rutin | | 1ad4 | -8.2 | 4urn | | -8.5 | | 5tcu | -8.0 | | 4c13 | | -10.9 | 5tw8 | | -9.3 |
|  | isorhamnetin 3-O-glucoside | | 1ad4 | -7.1 | 4urn | | -7.3 | | 5tcu | -7.2 | | 4c13 | | -9.1 | 5tw8 | | -8.7 |
| ***E. coli*** | isorhamnetin | | 1e8c | -8.6 | 4v53 | | -7.8 | | 6ntw | -7.4 | | 1kzn | | -8.1 | 5v7a | | -7.8 |
|  | p-coumaric acid | | 1e8c | -6.7 | 4v53 | | -5.4 | | 6ntw | -5.4 | | 1kzn | | -5.7 | 5v7a | | -5.3 |
|  | quercetin 3-O-glucoside | | 1e8c | -9.5 | 4v53 | | -8.3 | | 6ntw | -8.4 | | 1kzn | | -8.1 | 5v7a | | -8.8 |
|  | 3-O-Caffeoylquinic acid | | 1e8c | -8.7 | 4v53 | | -8.7 | | 6ntw | -7.8 | | 1kzn | | -7.7 | 5v7a | | -8.1 |
|  | 5-O-Caffeoylquinic acid | | 1e8c | -8.6 | 4v53 | | -8.6 | | 6ntw | -7.7 | | 1kzn | | -8.1 | 5v7a | | -8.2 |
|  | isorhamnetin 3-O-rutinoside | | 1e8c | -9.6 | 4v53 | | -8.4 | | 6ntw | -8.7 | | 1kzn | | -8.2 | 5v7a | | -8.0 |
|  | rutin | | 1e8c | -10.6 | 4v53 | | -8.7 | | 6ntw | -8.6 | | 1kzn | | -8.2 | 5v7a | | -8.1 |
|  | isorhamnetin 3-O-glucoside | | 1e8c | -9.4 | 4v53 | | -8.1 | | 6ntw | -7.8 | | 1kzn | | -8.0 | 5v7a | | -8.9 |
|  |  | |  |  |  | |  | |  |  | |  | |  |  | |  |

**Table S3.** Selected protein-ligand complexes for MM/PBSA binding free energy analysis based on molecular dynamics simulations.

| **Complex** | **Frames** | **VDWAALS** | **EEL** | **EGB** | **ESURF** | **GGAS** | **GSOLV** | **TOTAL** |
| --- | --- | --- | --- | --- | --- | --- | --- | --- |
| *E. coli MurE isorhamnetin_3-O-glucoside* | Average;SEM;SD | -30.92 (SD =6.88, SEM = 0.96) | -67.29 (SD =24.13, SEM = 3.38) | 79.62 (SD = 15.64, SEM = 2.19) | -5.79 (SD = 0.96, SEM = 0.13) | -98.21 (SD = 26.86, SEM = 3.76 | 73.83 (SD = 15.09, SEM = 2.11) | -24.38 (SD = 13.07, SEM = 1.83) |
| *E. coli MurE_rutin* | Average;SEM;SD | -52.2 (SD = 6.98, SEM = 0.98) | -44.15 (SD = 14.09, SEM = 1.97 | 72.26 (SD = 8.23, SEM = 1.15) | -7.88 (SD = 0.78, SEM = 0.11) | -96.35 (SD = 14.25, SEM = 1.99) | 64.39 (SD = 7.94, SEM = 1.11) | -31.96 (SD = 7.37, SEM = 1.03) |
| *Amylase_rutin* | Average;SEM;SD | -48.71 (SD = 3.24, SEM = 0.45) | -28.61 (SD = 3.95, SEM = 0.55) | 53.67 (SD = 3.17, SEM = 0.44) | -6.75 (SD = 0.36, SEM = 0.05) | -77.32 (SD = 5.58, SEM = 0.78) | 46.93 (SD = 2.99, SEM = 0.42) | -30.39 (SD = 3.83, SEM = 0.54) |
| *AChE_quercetin_3_O_glucoside* | Average;SEM;SD | -51.3 (SD = 3.85, SEM = 0.54) | -62.47 (SD = 7.41, SEM = 1.04) | 67.78 (SD = 4.63, SEM = 0.65) | -7.09 (SD = 0.18, SEM = 0.03) | -113.77 (SD = 7.95, SEM = 1.11) | 60.69 (SD = 4.56, SEM = 0.64) | -53.08 (SD = 5.19, SEM = 0.73) |
| *3djy_isorhamnetin_3_O_rutinoside* | Average;SEM;SD | -60.33 (SD = 3.69, SEM = 0.52) | -49.27 (SD = 9.5, SEM = 1.33) | 70.64 (SD = 5.25, SEM = 0.74) | -7.87 (SD = 0.27, SEM = 0.04) | -109.61 (SD = 10.79, SEM = 1.51) | 62.77 (SD = 5.15, SEM = 0.72) | -46.83 (SD = 6.69, SEM = 0.94) |
| *BChE_quercetin_3_O_glucoside* | Average;SEM;SD | -46.75 (SD = 3.51, SEM = 0.49) | -44.44 (SD = 14.26, SEM = 2) | 62.17 (SD = 6.93, SEM = 0.97) | -6.36 (SD = 0.2, SEM = 0.03) | -91.2 (SD = 13.67, SEM = 1.91) | 55.81 (SD = 6.92, SEM = 0.97) | -35.38 (SD = 7.58, SEM = 1.06) |
| *S. aureus MurE 3-O-caffeoylquinic_acid* | Average;SEM;SD | -36.34 (SD = 3.73, SEM = 0.52) | 72.84 (SD = 20.7, SEM = 2.9) | -67.15 (SD = 18.99, SEM = 2.66) | -6.29 (SD = 0.28, SEM = 0.04) | 36.5 (SD = 19.12, SEM = 2.68) | -73.44 (SD = 19.15, SEM = 2.68) | -36.93 (SD = 4.23, SEM = 0.59) |
| *S. aureus MurE_isorhamnetin 3-O-glucoside* | Average;SEM;SD | -46.9 (SD = 3.18, SEM = 0.45) | -47.68 (SD = 10.85, SEM = 1.52) | 65.24 (SD = 8.17, SEM = 1.14) | -6.87 (SD = 0.36, SEM = 0.05) | -94.58 (SD = 10.34, SEM = 1.45) | 58.36 (SD = 7.99, SEM = 1.12) | -36.22 (SD = 3.98, SEM = 0.56) |
| *E. coli 30S ribosome S3_quercetin 3-O-glucoside* | Average;SEM;SD | -25.79 (SD = 3.85, SEM = 0.54) | -55.92 (SD = 28.51, SEM = 3.99) | 63.99 (SD = 21.95, SEM = 3.07) | -4.69 (SD = 0.77, SEM = 0.11) | -81.71 (SD = 29.65, SEM = 4.15) | 59.31 (SD = 21.31, SEM = 2.98) | -22.4 (SD = 9.7, SEM = 1.36) |
| *Tyr_rutin* | Average;SEM;SD | -42.27 (SD = 3.52, SEM = 0.49) | -32.22 (SD = 18.55, SEM = 2.6) | 51.27 (SD = 13.93, SEM = 1.95) | -5.74 (SD = 0.4, SEM = 0.06) | -74.49 (SD = 19.36, SEM = 2.71) | 45.53 (SD = 13.64, SEM = 1.91) | -28.95 (SD = 6.75, SEM = 0.95) |
| *E. coli Transpeptidase_isorhamnetin 3-O-glucoside* | Average;SEM;SD | -30.81 (SD = 6.04, SEM = 0.85) | -22.1 (SD = 14.45, SEM = 2.02) | 44.03 (SD = 11.38, SEM = 1.59) | -4.48 (SD = 0.88, SEM = 0.12) | -52.91 (SD = 18.7, SEM = 2.62) | 39.55 (SD = 10.64, SEM = 1.49) | -13.36 (SD = 8.7, SEM = 1.22) |


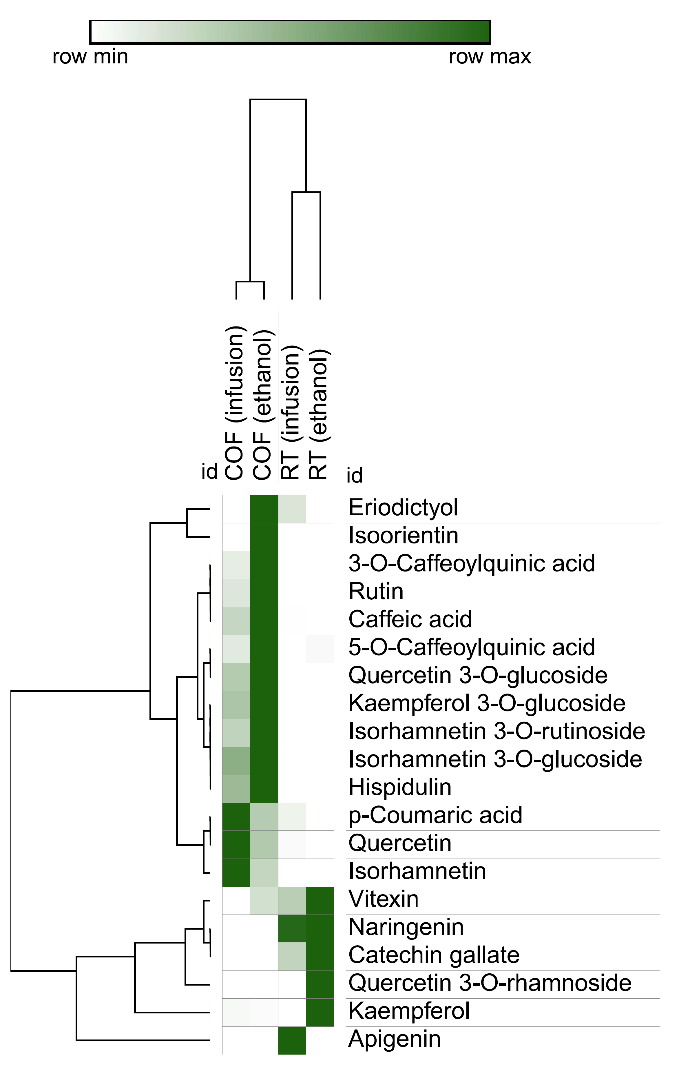


Figure S1. Heatmap of scaled quantitative data, with samples (both columns and rows) arranged by HCA (Spearman cluster agglomeration method). The intensity of the green color indicates the abundance of the compound in the samples.
